# Advances in Optical Sensors for Persistent Organic Pollutant Environmental Monitoring

**DOI:** 10.3390/s22072649

**Published:** 2022-03-30

**Authors:** Fabrizio Caroleo, Gabriele Magna, Mario Luigi Naitana, Lorena Di Zazzo, Roberto Martini, Francesco Pizzoli, Mounika Muduganti, Larisa Lvova, Federica Mandoj, Sara Nardis, Manuela Stefanelli, Corrado Di Natale, Roberto Paolesse

**Affiliations:** 1 Department of Chemical Science and Technologies, University of Rome “Tor Vergata”, 00133 Rome, Italy; crlfrz01@uniroma2.it (F.C.); gabriele.magna@uniroma2.it (G.M.); mrtrrt02@uniroma2.it (R.M.); francesco1.pizzoli@gmail.com (F.P.); mounika.muduganti@students.uniroma2.eu (M.M.); federica.mandoj@uniroma2.it (F.M.); nardis@scienze.uniroma2.it (S.N.); manuela.stefanelli@uniroma2.it (M.S.); paolesse@uniroma2.it (R.P.); 2 Department of Science, Roma Tre University, Via della Vasca Navale 84, 00146 Rome, Italy; marioluigi.naitana@uniroma3.it; 3 Department of Electronic Engineering, University of Rome “Tor Vergata”, 00133 Rome, Italy; lorena.dizazzo@uniroma2.it (L.D.Z.); dinatale@uniroma2.it (C.D.N.)

**Keywords:** persistent organic pollutants, chemical sensors, ecological monitoring, health risks assessment, optical transduction, multisensor analysis, biological sensors

## Abstract

Optical chemical sensors are widely applied in many fields of modern analytical practice, due to their simplicity in preparation and signal acquisition, low costs, and fast response time. Moreover, the construction of most modern optical sensors requires neither wire connections with the detector nor sophisticated and energy-consuming hardware, enabling wireless sensor development for a fast, in-field and online analysis. In this review, the last five years of progress (from 2017 to 2021) in the field of optical chemical sensors development for persistent organic pollutants (POPs) is provided. The operating mechanisms, the transduction principles and the types of sensing materials employed in single selective optical sensors and in multisensory systems are reviewed. The selected examples of optical sensors applications are reported to demonstrate the benefits and drawbacks of optical chemical sensor use for POPs assessment.

## 1. Introduction

Persistent organic pollutants (POPs) are a class of substances of the most critical environmental challenges of today because they are organic compounds characterized by resistance to degradation, ability to bioaccumulate in the tissues of organisms, and significant adverse impacts on the health of humans and the environment. Furthermore, as POPs are resistant to degradation and remain in the environment for a long period, they can be transported far from the place of production, leading to their distribution across the globe, including the contamination of remote regions far from the place of their manufacture, use and disposal.

The Stockholm Convention [1] in 2001 defined a starting list of POPs, whose production and use were recommended to be stopped and strongly reduced. In this list, twelve compounds, named “the dirty dozen”, were included, grouping organochloride pesticides and industrial compounds such as PCBs. Other compounds have been added later to this list, such as organophosphorus (OPs) pesticides, flame retardants, polyaromatic hydrocarbons (PAHs) and, recently, per- or poly-fluoroalkyl compounds (PFAS). However, this list is nonexhaustive, considering the huge number of chemicals synthesized and used for several purposes.

The sources of POPs are different. They can be intentionally spread into the environment, for example pesticides needed to control pests to prevent disease and to improve agriculture production. Other chemicals have been intensively produced in various industrial processes, for example PCB, which is used as oils, as dielectric and cooling fluids in capacitors and transformers, and which have been unintentionally introduced in the environment as a result of spills and evaporation at the end of the life of several devices containing PCBs.

POPs are a concern, as they are persistent in the environment; thus, they can be easily spread far from their original site. These compounds can bio-accumulate in the food chain due to their lipophilic properties, and they show adverse effects ranging from acute to chronic toxicity [2]. Different pathologies have been reported to be correlated with POPs exposure, such as cancer, allergies and hypersensitivity, damage of the central and peripheral nervous systems, reproductive disorders, and disruption of the immune system [3]. The characteristic of POPs to diffuse in locations far from their release into the environment makes it impossible for a single government to act to adopt strategies to protect the population or environment from POPs, making these compounds a worldwide global problem. From this point of view, the European Union has banned the use of POPs via Regulation 2019/1021 [4], which implements the actions necessary to eliminate their production and commercialization.

POPs are generally present in low amounts, and for this reason, the ability to detect POPs sensitively and selectively is crucial information, and there is a pressing need to follow the presence of POPs in water and environmental contaminations, as well as in food chains.

There has been a huge effort to find and optimize analytical tools for monitoring POPs, with chromatographic techniques coupled to mass spectrometry widely used for the qualitative and quantitative detection of POPs. These analytical laboratory techniques can satisfy the sensitivity and selectivity requirements, but they suffer from the need to necessitate skilled and trained personnel and expensive instruments for analysis. In addition, time-consuming extraction and preconcentration techniques are always necessary. Moreover, these techniques are unsuitable for remote monitoring of water and environmental pollution.

For this reason, the need for cost-effective, flexible, and reliable methods is still unmet. The development of chemical sensors has been conceived to offer a solution that can bypass the limitations of the classical analytical protocols.

While different transduction mechanism can be exploited to develop chemical sensors, optical sensors are the most promising to satisfy the sensitivity requirements necessary for POPs detection.

Depending on the optical features considered, it is possible to exploit the optical variation of the target POP, such as the case of Raman spectroscopy, or to use the variation of the optical properties of an appropriate receptor upon interaction with the target pollutant, which is usually exploited. Although some examples of naked eye detection of colorimetric systems have been reported, fluorescence-based sensors are in this case the most popular devices because of the high sensitivity requirements.

Sensor selectivity is another important aspect for POPs detection, because of the large number of compounds to be analyzed. This makes the design of appropriate receptors difficult and renders it impossible to achieve the detection of all the different kinds of POPs. This route is viable only when few analytes should be detected, while it is practically impossible in the case of complex mixtures, where a huge number of chemicals characterize the chemical matrix. For this reason, in the case of complex matrices of unknown composition, such as POP-polluted water samples, the application of highly selective sensors is complicated by the large number of devices necessary to detect the different analytes potentially present in the target matrix.

The sensor array approach will offer a solution to these difficulties, both reducing the synthetic effort for the selection of receptors and allowing for the possibility to classify the diversity of the target analytes [5]. Sensor arrays can be applied not only to distinguish among target POPs, but also to quantify their presence, after preliminary calibration steps.

In this review, we want to summarize the recent (over the last five years, from 2017 to 2021) developments in the field of POPs detection by the different kinds of optical sensors, according to the different transduction mechanisms exploited. 

## 2. Optical Sensors for POPs Assessment 

Optical devices transform optical phenomena changes, arising as a result of the interaction between the analyte and the receptor part [6] into digital/electrical signals employing different transduction techniques to yield analyte information [7]. Optical chemical sensors may be classified according to the type of optical properties that have been applied: *absorbance* (measured in a transparent medium and caused by the absorptivity of the analyte itself or by a reaction with some suitable indicator); *reflectance* (measured in non-transparent media, usually using an immobilized indicator); *luminescence* (estimated by the intensity of light emitted by a chemical reaction in the receptor system); *fluorescence* and *phosphorescence* (both measured as the positive emission effect caused by irradiation). The wavelength of emitted irradiation can be modulated by *scattering* (caused by particles of different sizes present in analyzed sample), *refraction* (expressed through the variation of refraction index upon the variation of analyzed sample composition; also includes a surface plasmon resonance effect), or *reflectance* (measured in non-transparent media). In addition, selective quenching of fluorescence, or *optothermal effect* based on a measurement of the thermal effect caused by light absorption, may be the basis of such devices. Generally, the employed optical technique, type of the recognition element, and the sensing platform construction (including transductor and reagents involved) can be used for optical chemical sensors classification [8].

The most widely used technique employed in optical chemical sensors for POPs remains fluorescence, but sensors based on other spectroscopies as well as on optical parameters, such as refractive index (SPR) and reflectivity (SERS) systems, colorimetric sensors, biosensors and multisensor systems, have also been developed. A brief overview on applications of spectroscopy technology to trace the analysis of persistent organic pollutants was previously published by Wang, Pang, and Zhou [9]; the techniques for the detection of dioxins, furans and related compounds based on optical and spectroscopic methods were reported by Patrizi et al. [10]. In [11], the recent updates on the strategies for organochlorine pesticides SERS detection were provided. In [12], the detection methods of POPs in food, along with their contamination sources and effects on health, are discussed. 

**Scheme 1:**
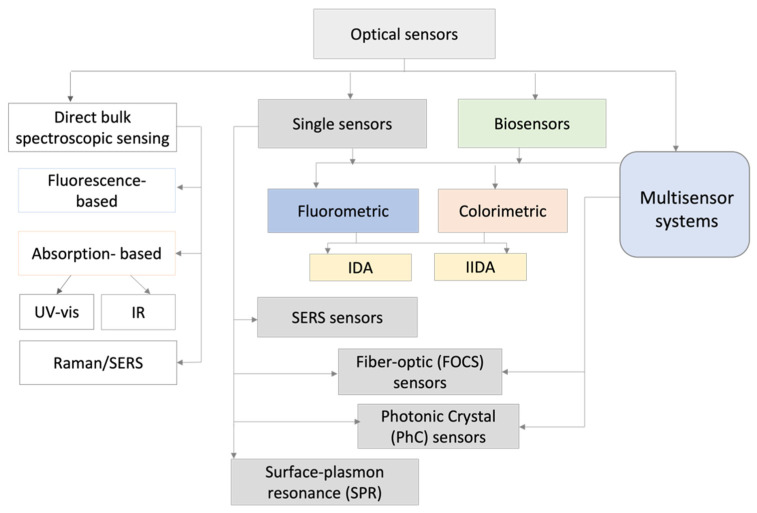
The structure of the present review on optical chemical sensors for POPs assessment.

Despite the above-mentioned publications, dedicated to the selected applications of some specific types of optical chemical sensors for some types of organic polluting compounds, until now, no summarizing review fully representing the actual achievements in the field of optical chemical sensors for POPs detection in the environment has been published. In this regard, in the following sections, an overview on POPs optical sensors developed over the last five years, according to sensing principles, construction/transduction, and applications, including the brief description of sensing mechanisms, is provided. The structure adopted for this review is shown in Scheme 1, while the information on the employed sensory material, transduction principle, the specific POPs compounds that can be detected, and other details, such as the limit of detection, linear response concentration range, and application fields of both single optical sensors and multisensor arrays, have been summarized in Table 1. Although they employ different transduction mechanisms, we have placed the optical biosensors as a separate group, since they involve biological species as specific receptors inducing chemical processes.

## 3. Fluorometric Sensors

**Scheme 2:**
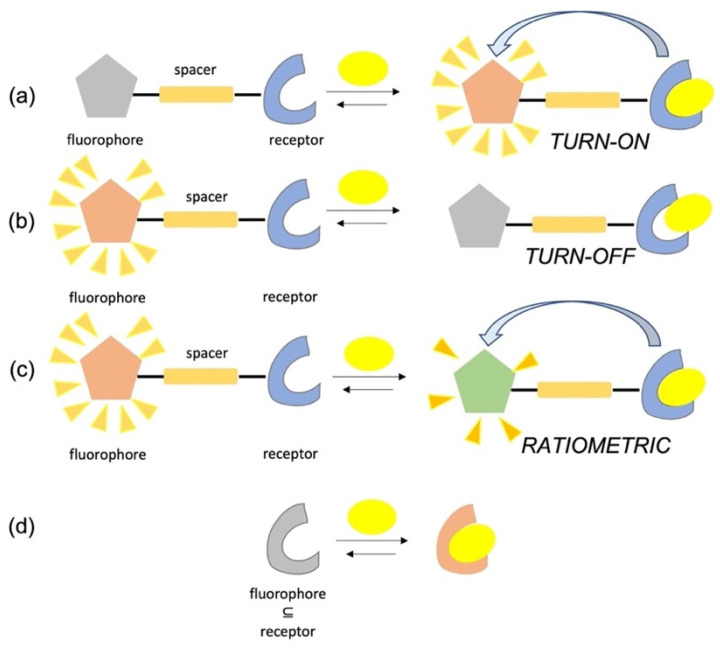
Schematic representation of the main designs and working mechanisms of fluorescent sensors: (**a**) “turn-on”, (**b**) “turn-off”, (**c**) ratiometric, and (**d**) the easier system where the receptor is itself fluorescent. Of course, if the recognition unit is fluorescent, an easier sensing system is also exploitable (**d**).

Supramolecular fluorescent sensors [13] are typically based on the ‘‘fluorophore–spacer–receptor unit” layout, where the light-emitting unit is connected to the analyte binding moiety (receptor) by a linker that allows the fluorophore to produce detectable readout(s) when the selective complexation of the target analyte occurs (Scheme 2a).

The interactions between analytes and receptors can variously affect the fluorophore emission profiles and can be triggered by different mechanisms such as fluorescence resonance energy transfer (FRET), photo-induced electron transfer (PET), intramolecular charge transfer (ICT), chelation-induced enhanced fluorescence (CHEF), aggregation-induced emission (AIE) and others [14]. Since the energy or electron transfer following the analyte binding can lead to an enhancement, quenching of fluorescence emission intensities or to blue/red shifts of maxima, fluorescent sensors are usually divided into three classes, i.e., ‘‘turn-on”, ‘‘turn-off”, and ratiometric. A schematic illustration of their operating mechanisms is depicted in Scheme 2a–c. As far as the ratiometric sensors are concerned, different changes in the intensities of two or more fluorescence bands upon analyte addition are monitored, and the measurement of their relative ratio allow for the internal self-calibration, resulting in high detection accuracy. As a general mindset, ‘‘turn-on” and ratiometric sensors are preferred over ‘‘turn-off” probes since they combine higher sensitivity and lower probability to give false responses in respect to the latter. Additionally, turn-off sensors cannot be used in environments with high background emission, such as cellular imaging. 

**Scheme 3:**
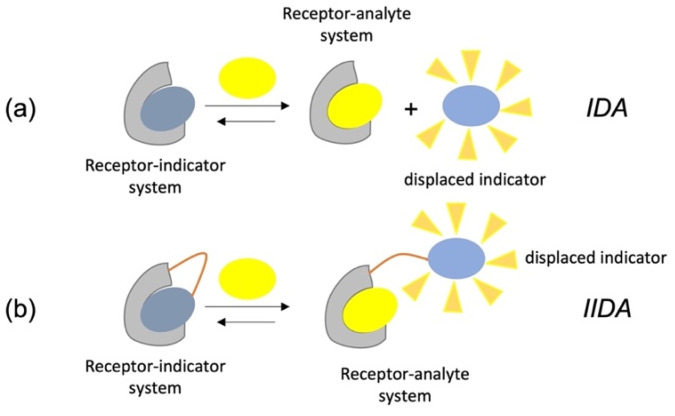
Schematic representation of (**a**) the Indicator Displacement Assay (IDA) and (**b**) the Intramolecular Indicator Displacement Assay (IIDA) used in fluorometric sensors.

As an alternative to the direct sensing, optical sensors can be designed on competitive binding of multiple molecules for the receptor. This approach, frequently used also in biochemistry, is known as an “indicator displacement assay” (IDA) and involves the competition between an indicator and the target analyte for the binding site of a receptor. Typically, a receptor–indicator system is used as a sensing unit at the first stage of the assay. When the analyte is added, it is bound from the receptor triggering to the release of the indicator into solution. The difference in the optical properties for the free and bound indicator molecule give the signal variation useful for the analytical quantification. Of course, in fluorometric sensor development, the indicators are the fluorescent signaling units. Further, a slight variation of this approach is represented by the IIDA, i.e., intramolecular indicator displacement assay, where the indicator is covalently linked to the receptor. Schematic representation of these two analytical approaches is reported in Scheme 3.

Concerning the detection of POPs using fluorometric sensors, several examples have been recently discussed in the literature. Many analytes reported are in general detected by other methods, including chromatographic separation coupled to mass spectrometry or even electrochemical devices. Due to their easy operation, fast response and, above all, their high sensitivity, fluorescent methods are continuously developed and explored. Hereafter, we have focused on the detection of three representative classes of persistent toxicant analytes by fluorescence reported in the literature in the last five years. 

### 3.1. PFAS Optical Detection

Per- and polyfluoroalkyl substances (PFAS) are a family of organic anionic compounds extensively employed in consumer product manufacturing and industrial processes [15]. Due to their exceptional stability in natural environment, bioaccumulation, and high toxicity, PFAS have emerged as global persistent organic pollutants. The high strength of C-F carbon makes PFAS thermodynamically stable and resistant to typical degradation pathways, such as biodegradation and photolysis. Usually, PFAS are found as negatively charged anions, but depending on pH and other functional groups, cations and zwitterions also exist, which can affect transportation and sorption. These varied physicochemical properties make PFAS difficult to detect as a class. Specific attention has been given to perfluorooctane sulfonate (PFOS) and perfluorooctanoic acid (PFOA) in which its potential ecotoxicity is well known by now. Therefore, the development of simple, rapid, and sensitive detection methods is of great importance for PFAS monitoring and environmental health risk appraisal.

**Figure 1:**
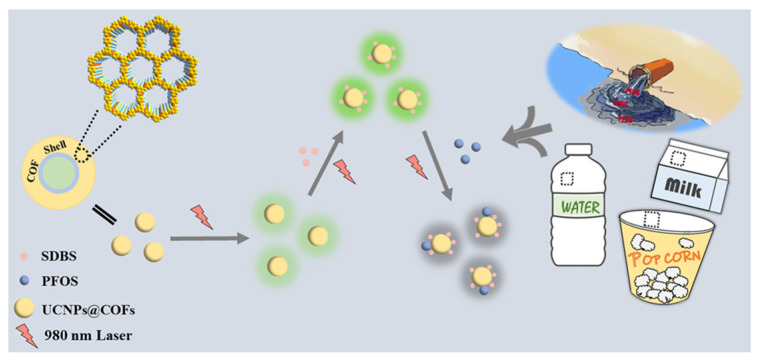
Sensing system based on the fluorescence quenching of UCNPs@COFs by PFOS in the presence of SDBS. Reprinted from [16].

Recently, different fluorescent nanomaterials have been used to construct fluorescent probes to employ for the monitoring of PFOS and PFOA in aqueous samples. Li et al. [16] proposed a highly sensitive fluorescence approach for sensing ultra-trace PFOS, based on a novel fluorescent probe, integrating the advantages of upconversion nanoparticles UCNPs and covalent organic frameworks COFs. Such a fluorescent probe was synthesized via the solvothermal growth of COFs on the surface of UCNPs (denoted as UCNPs@COFs). The fluorescence response of UCNPs@COFs to PFOS could be significantly enhanced by anion surfactants. From the quenching mechanism, it was deduced that the layer of COFs enriched more PFOS molecules into its pore channel, in the presence of sodium dodecyl benzene sulfonate (SDBS), and the highly electronegative PFOS caused the fluorescence quenching of UCNPs@COFs, as shown in Figure 1. With the sensing system, the amount of PFOS spiked in tap water and food packing was successfully analyzed. 

**Figure 2:**
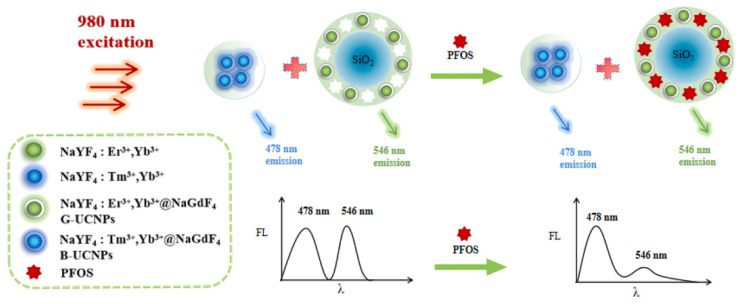
Schematic illustration of the upconversion molecular imprinted ratiometric probe. Reprinted from [18].

Lin et al. [17] proposed a fluorescent sensor system based on a different nanomaterial such as carbon dots. Nitrogen-doped carbon dots (NCDs) were prepared through a hydrothermal method via vitamin B1 as a precursor and triethylamine as a doping agent. The authors highlighted how NCDs demonstrated photoluminescence emission with a quantum yield of 12%, robust resistance against photobleaching, solution pH values, temperature, and ion strength. The nanoparticles responded selectively and sensitively even to trace concentrations of perfluorooctane sulfonate (PFOS) through electrostatic interactions between PFOS and NCDs. This was accompanied by the aggregation of NCDs to yield enhanced fluorescence. Moreover, the nanoprobe has been shown high selectivity for PFOS even in the presence of other common ions such as metal ions, anions, and structural analogues such as surfactants. Satisfactory results were achieved for determination of PFOS in spiked real water samples. Recently, Tian and coworkers [18] have proposed a molecularly imprinted near-infrared excitation ratiometric fluorescent probe for the detection of PFOS, based on a unique optical nanomaterial, such as lanthanide-doped upconversion nanoparticles (UCNPs). The nanoprobe was constructed by physically mixing two different UCNPs emitting green and blue fluorescence, as response signal and reference signal, respectively. The sensing process was achieved through the selective recognition of specific cavities in the probe surface with analyte, accompanied by fluorescence quenching due to the PET effect between upconversion materials and PFOS, as shown in Figure 2. 

In comparison with conventional fluorophores, UCNPs have the advantages of deep penetration and low background, which are suitable for application in environmental and biological analysis. For the detection and absorption of pollutants, an efficient molecular recognition, in aqueous media, has been also demonstrated by organic conjugated materials.

**Figure 3:**
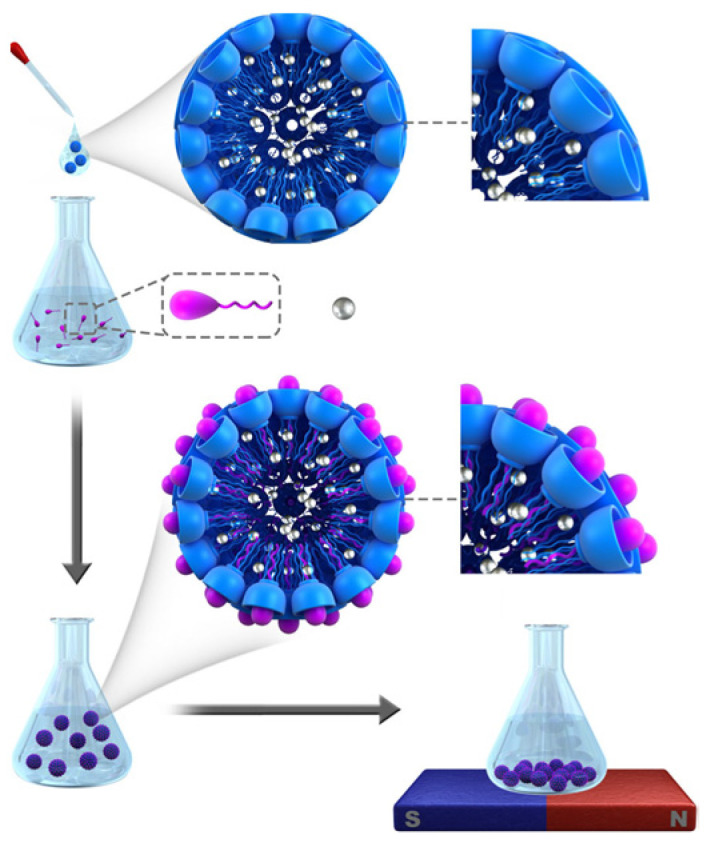
Illustration of the absorption and magnetic separation procedure of PFOS and PFOA by the MNP@GC5A-12C nanoparticle. Reported from [19].

Zheng and coworkers [19] reported the nanomolar binding of two different guanidinocalix[5]arenes (GC5A-6C and GC5A-12C, having 6 and 12 carbon atoms in each alkyl chains at the lower rim of the macrocycles, respectively) toward PFOS and PFOA. Taking advantage of the strong recognition and supramolecular assembly, they achieved not only sensitive and quantitative detection of PFOA and PFOS, in tap and lake water, through the IDA strategy, but also the efficient removal of them by the hybrid nanoparticle. By co-assembling iron oxide nanoparticle (MNP) with the amphiphilic guadinocalix[5]arene (MNP@GC5A-12C), the authors were able to efficiently remove PFOA and PFOS from contaminated water via a simple magnetic absorption and filtration procedure, as shown in Figure 3.

**Figure 4:**
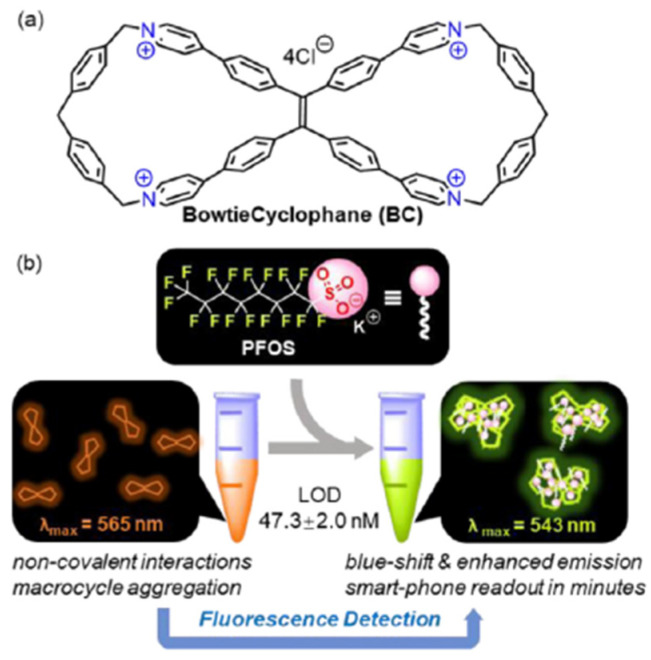
The (**a**) chemical structure and (**b**) the schematic representation of the development of BC as a fluorescence sensor for PFOS detection. Reported from [20].

Supramolecular chemistry concepts have been also exploited by Lei et al. [20] that presented BowtieCyclo (BC), a water-soluble, fluorescent macrocycle, as a novel PFOS sensor, as shown in Figure 4. Structurally, BC is a symmetrical tetra-cationic figure-of-eight macrocycle, which combines a tetraphenylethylene (TPE) core, and pyridinium cyclophane scaffolds on both sides. The addition of PFOS can induce aggregation of BC, triggering significant changes in both fluorescence intensity and color. This system represents the first aggregation-induced emission (AIE)-based macrocyclic fluorescence sensor for PFOS detection in aqueous solution. The authors showed how fluorochromism-based PFOS detection is visualizable and quantifiable using a smartphone.

Recently, Zhang et al. [21] chose perylene diimide derivative (PDI), a different organic conjugated substrate, to develop a selective and simple strategy for PFOS detection. They designed and synthesized a cationic PDI derivative, N,N′-bis((1-pyridium)ethyl)-3,4,9,10-perylenetetracarboxylic acid diimide (PDI-Pyr) as fluorescent probe; they included the pyridinium moiety that enhances the water solubility of PDI and promotes a good dispersed state in aqueous solution. In the presence of PFOS, PDI aggregates are formed under electrostatic and hydrophobic interactions between PDI-Pyr and PFOS. Since this process was accompanied by sharp quenching of fluorescence, the developed system can be used for PFOS detection in a “turn-off” mode.

### 3.2. Aromatic Toxicant Compounds

**Figure 5:**
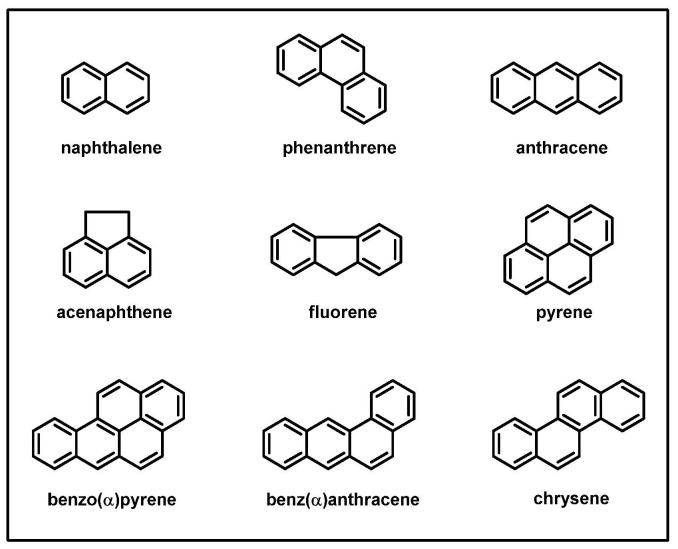
Representation of the structures of the main PAHs.

Since many aromatic contaminants exhibit strong and characteristic fluorescence emission, fluorometric sensing systems have been abundantly reported in the literature for the detection of different classes of aromatic POPs, especially for PAHs in different matrices. This class originates mostly from incomplete combustion of petroleum products and include a series of compounds having a variable number of fused aromatic rings without heteroatoms or peripheral groups, as shown in Figure 5. 

Once produced, PAHs are incorporated into different environmental scenarios by both natural and anthropogenic events, jeopardizing all living beings due to their cancerogenic properties [22]. Accordingly, their detection is an imperative task to comply and, on the subject, some recent studies exploiting fluorometric sensors have been reported by Bortolato and coworkers. In a first explorative paper, they showed the potentiality of bio-inspired water-soluble copolymers of proper composition based on thymine and anionic groups to detect benzo[a]pyrene (BaP) in water by enhancing its native luminescence [23]. The polymeric system has demonstrated to be able to set non-covalent interactions with the target contaminant both in solution and once immobilized on solid PET slides. On these foundations, the same group constructed a sensitive and versatile fluorescent sensor with eco-scale due to the biodegradability of the sensitizer used, widening the application on pyrene (Pyr) [24]. Both analytes have been quantified at the required levels in drinkable water, being 0.11 and 0.06 ng/mL for BaP and Pyr, respectively. Further, they are simultaneously detected with high relative selectivity due to the use of multivariate analysis, i.e., the second-order calibration based on the parallel factor analysis (PARAFAC) algorithm to process second-order excitation–emission fluorescence data.

Beside these two toxicants, other congeners and related suspected metabolites have been identified and quantified in human breast milk by a supramolecular “turn-on” fluorometric system developed by Levine’s group [25]. The studied system was based on a γ cyclodextrin-dye complex where fluorescence emission signals increase due to energy transfer from the bound PAH to high quantum yield fluorophore, promoted by cyclodextrin macrocycle. Lipophilic medium composition helps the inclusion of the analytes by forming hydrophobic micelles with high affinity for the aromatic platforms and consequently favoring their interactions with the encapsulated dye. A wide collection of aromatic analytes generally found in different biological fluids have been selected, and their ability to “turn-on” the fluorescence emission for three different dyes (i.e., rhodamine, coumarin and BODIPY derivatives) was investigated, with the γ cyclodextrin present or not. For many combinations of analyte–fluorophore, the addition of a cyclodextrin receptor had no significant impact on the observed energy transfer efficiencies, testifying the formation of strong host–guest complexes between the target aromatic platforms and the emissive dyes. These high affinities enabled the detection of PAHs at concentrations as low as 0.17 μM. The use of array-based statistical analysis (LDA, linear discriminant analysis) allowed for the selective differentiation of the tested analytes, even among those structurally analogues. This was practicable due to the different “fingerprint” exhibited in the fluorescence spectra for each analyte–fluorophore combination.

Other important aromatic compounds of high environmental impact to monitor are benzene derivatives containing halogens and amino groups. Polychlorinated aromatic compounds are largely used both in industrial and agricultural applications, where they are administrated as pesticides. They are released in soil and diffuse into the groundwater, reaching the living organisms where they tend to bioaccumulate and cause serious health problem [26]. Fluorometric detection of these compounds has been recently performed by Cheng and coworkers, exploiting the quenching in the red-emission signal of a Eu3^+^-MOF assembled system in organic solutions containing polychlorinated benzenes with a variable numbers of Cl atoms [27]. The system displayed well-resolved luminescence of the f–f transitions (λ_ex_ = 294 nm) with four peaks in the red region, centered at 592, 616, 652 and 704 nm, respectively, that change upon analyte addition. The authors found that the increasing number of chlorines more significantly affected the luminescence intensity of the system that accordingly resulted decreased. More importantly, the lanthanide-MOF was found to be highly selective and sensitive for the detection of 1,2,4-trichlorobenzene (1,2,4-TCB) in mM DMF solutions containing different polychlorinated benzenes and organic small solvent molecules, such as hexane or alcohols. Of course, the quenching effect was higher in the case of aromatic compounds tested. The sensing mechanism was studied in detail. Basing on structural parameters of both substituted benzenes and pores of the Eu3^+^-MOF, analytes can only interact with the surface, being hampered the entering of benzene rings the channels of the sensing material. Conversely, a direct energy transfer between guest molecules and ligands was ruled out by DFT calculations. More reasonably, the quenching process was due to a competition of the absorption of the excited light between the aromatic analytes and ligands. The reported experiments showed that polychlorinated benzenes filtered the light absorbed by the ligand L, with the consequent decreasing in the total energy transfer from L to lanthanide ions, reflecting in a lowered luminescence.

Amines are well-known harmful pollutants causing serious damages to respiratory, central nervous system and cardiovascular systems or even tumors. An efficient sensor for primary aromatic amines (PAAs) detection among various amines has been reported by Zhong et al. [28]. The fluorescent system combined two different molecular components, namely the fluorescent 2,1,3-benzothiadiazole (BT) derivative inserted into a 2D porous covalent triazine framework (CTF). BT-based molecules are known as ACQgens, i.e., aggregation-caused quenching luminogens, whose fluorescence can be maintained by their covalent immobilization into a molecular scaffold hampering their aggregation. The prepared material possesses absolute higher quantum yield in comparison with the BT-based monomer, being 33.6% and 8.32% upon excitation of 416 and 374 nm, respectively. This is due to the rigidity effect given by the incorporation into the nanosheets, where ACQ gens were also largely dispersed, thus being prone to sense the target analytes. These fluorophores are electron-deficient, and they are good candidates for the recognition of electron-rich amines that can perturb their photoluminescence properties. The excellent water stability of the porous and highly fluorescent material obtained prompted the authors to explore its sensing ability toward nine classes of amines, selected among the aliphatic, aromatic, heterocyclic and quaternary ammonium salt. The high fluorescence quenching efficiency of the developed sensors was found for the three primary aromatic amines tested (phenylamine, PA; p- phenylenediamine, PDA; 1-naphthylamine, NPA). For these analytes, LOD values of 11.7, 1.47 and 26.2 nM for PA, PDA and NPA, respectively, were estimated, with the first two being much lower than any reported chemosensor to date. Combining experimental findings and theoretical calculations, the selectivity and sensitivity for PAAs was ascribed to the static quenching process caused by the H bonds formation between aromatic amines and the material forming the ground state non-fluorescent complex.

### 3.3. Fungicide and Pesticide Assessment

**Figure 6:**
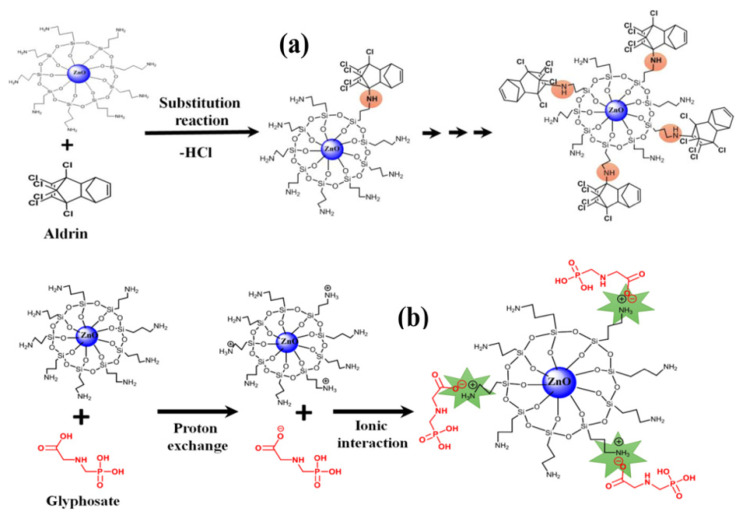
Schematic representation of ZnO QDs interaction (**a**) Aldrin and (**b**) Glyphosate. Reported from [30].

Arvand et al. [29] explored the possibility of utilizing optical biosensors to detect the presence of Edifenphos (EDI), an extremely toxic fungicide that is widely administrated in agriculture. The authors utilized an aptasensor developed by immobilizing an ssDNA aptamer for EDI on water-soluble L-cysteine-capped ZnS quantum dots (QDs). Mixing graphene oxide (GO) sheets with the aptamer-QDs enables a fluorescence resonance energy transfer from the QDs to the GO sheets, which in turn quenches the fluorescence yield of QDs. Restoring of fluorescence occurs in the presence of EDI that replaces GO interrupting the ongoing FRET mechanism. This GO-based aptasensor under the optimum conditions exhibited excellent analytical performance for EDI determination, ranging from 0.5 to 6 μg L^−1^, with an estimated detection limit of 0.13 μg L^−1^. Finally, the aptasensor exhibited excellent selectivity toward EDI compared to other pesticides and herbicides, with similar structures such as diazinon, heptachlor, endrin, dieldrin, butachlor and chlordane. Sahoo and coworkers fabricated a QDs-based optical sensor capable of detecting several pesticides at concentrations up to 4 ppm [30]. The authors selected four pesticides, namely aldrin, tetradifon, glyphosate, and atrazine, which are widely used in agriculture. ZnO QDs were prepared by sol–gel technique and coated with 3-aminopropyltrimethoxysilane (APTES), in order to create a shell preventing the aggregation of the QDs and their decomposition in H_2_O. The use of QDs led to two main advantages: interesting optical properties compared to the equivalent bulk structure (for example a large visible emission), and an enormous surface–volume ratio, as shown in Figure 6. Furthermore, this material can be used both for detection and remediation since pesticides are degraded by the catalytic activity of QDs. When excited with radiation at 340 nm, the ZnO QDs show a fluorescence peak at 525 nm. Due to dynamic quenching, this peak is damped to different degrees depending on the pesticide, allowing for discrimination. By measuring fluorescence responses at different concentrations of analytes, the authors assumed that the amine groups of the QDs give nucleophilic substitution toward good leaving groups of the pesticides (for example Cl ions). Therefore, from the number of leaving groups of the pesticide, it was possible to estimate the strength of the bond.

Time-resolved fluorescence decay measurement showed that the fluorescence decay depends on the concentration of pesticide. The authors found longer fluorescence lifetimes increased with the strength of the analyte-QDs binding. This latter aspect contributed to the detection and discrimination of the different pesticides.

### 3.4. Antibiotics Detection with Fluorimetric Optodes

**Figure 7:**
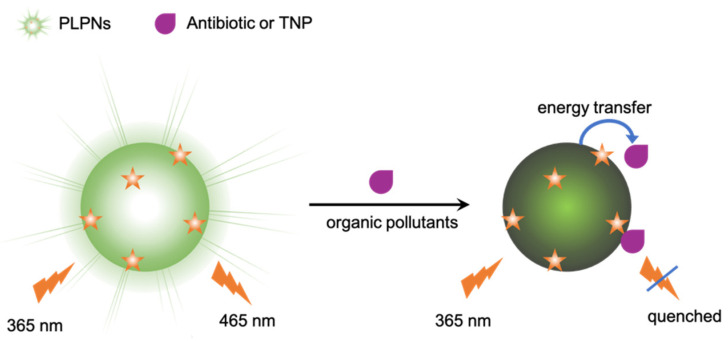
Schematic representation of PLNPs, adopted from [31].

The extensive use of antibiotics for treatment of bacterial infections both in animals and humans, represents a critical concern for environmental, food and health safety; thus, they are regarded as a class of important organic pollutants. Different nanomaterials have been proposed to solve this problem: Hu and coworkers [31], to find stable nanomaterials in water and, inspired by upconversion nanoparticles as alternatives to conventional luminescent bioprobes, have developed a new method for the synthesis of persistent luminescence nanoparticles (PLNPs). These light–light transformation and energy-saving materials can store absorbed light energy and release it as luminescence with a lifetime in the order of several hours, avoiding background interference with reduced light scattering and less autofluorescence. The authors constructed a label-free sensor whose luminescence can be quenched by antibiotics and nitroaromatic compounds, allowing for a qualitative and quantitative analysis, also in real samples. The Sr_2_Al_14_O_25_:Eu2^+^, Dy3^+^ nanoprobes were prepared by a hydrothermal–coprecipitation method and were characterized by transmission electron microscopy and dynamic light scattering to determine their shape and hydrodynamic diameter. Their photoluminescence properties were investigated: a maximum intense peak at 468 nm was obtained when the excitation wavelength was set at 370 nm with excellent long-lasting luminescence without further excitation (6 h), as shown in Figure 7.

The stability of the nanoprobes was investigated at different pH and in the presence of several ions, proving that they can work in different conditions with a PL intensity almost constant from pH 3 to 10 and in high ionic strength environments. The obtained long persistent luminescence nanoparticles were then successfully applied to the detection of antibiotics (as nitrofurazone) and nitroaromatic compounds (2,4,6-trinitrophenol, TNP) in real samples and test papers, with LOD values of 5 and 10 nM, respectively.

**Figure 8:**
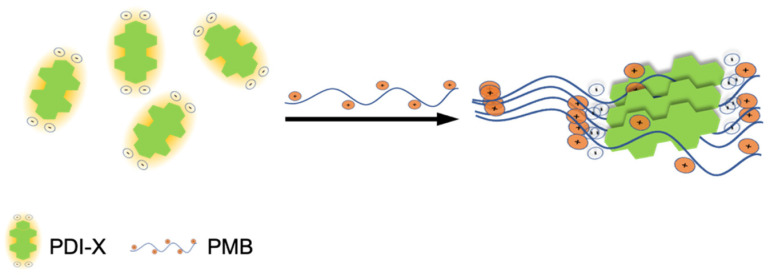
Schematic representation of PDI based sensors, adopted from [32].

Yao and coworkers [32], among various antibiotics families, focused their attention on berberine chloride (BBC), a natural isoquinoline alkaloide contained in many medicinal plants, exhibiting multiple pharmacological and biological properties, whose abuse can cause bacterial resistance and other adverse effects. In order to detect its presence, they developed fluorescent sensors based on water-soluble PDI derivatives prepared via one-step reactions between 3,4,9,10-perylenetetracarboxylic dianhydride and four amino acids (X = Asp, Glu, Ala and CysA), as shown in Figure 8. Among the PDI-X, the most promising PDI-Asp was chosen for further investigation: the system, based on the formation of supramolecular self-assemblies between PDI-Asp and BBC, is characterized by a decrease in the luminescent intensity of PDIs at 546 nm at increasing concentrations of antibiotic. The authors obtained a low-cost probe, characterized by rapid response, excellent water solubility, ease of observation and operation, with a detection limit of 28 nmol/L.

Based on the same precursor, Zhang et al. [21] has satisfied to rich a LOD as low as 18.5 nmol/L for the detection of Polymyxins B (PMB), a natural cyclic decapeptide antibiotic active against Gram-negative pathogens [33]. Even in this case, the formation of supramolecular aggregates of the dye, promoted by the antibiotic through the cooperation of π–π interactions and leading to a quenching of the PDI fluorescence, allowed for the realization of a sensitive and selective “turn-off” sensor (see Section 3.1). Taking into account that the quenching reaction begins immediately after mixing PDI and PMB and finishes within 1 min, the fabrication of test strips could be applied for the rapid determination of PMB in real samples.

High selectivity and high sensitivity for antibiotics have also been obtained by Fang and coworkers [34]. The researchers developed a method for the in-trace detection of ribaverin (RBV), an antiviral drug that can accumulate in animal bodies leading to several adverse effects including carcinogenicity and teratogenicity. For this purpose, they proposed a selectivity-enhanced ratiometric fluorescence imprinted sensor based on synergistic effect of covalent and non-covalent recognition units by coupling boric acid-functionalized lanthanide metal organic framework (BA-LMOFs) with a molecularly imprinted polymer (MIP). The BA–LMOFs@MIP and BA-LMOFs@NIP (nonimprinted polymer) were prepared as reported in the literature, in the presence and in the absence of RBV, and then their fluorescence and absorption behavior were investigated. BA–LMOFs@MIP exhibits dual emission peaks at 363 and 618 nm, confirming that the luminescence of Eu3^+^ is not affected by the coupling with boric acid. In the presence of RBV, the fluorescence intensity at 363 nm was increased, while the emission at 618 nm was quenched, with a more conspicuous response for the structure bearing the imprinted polymer. Selectivity, reusability, and stability of the sensor were then investigated, allowing the authors to report a wide linear range for RBV from 25 to 1200 ng/mL with a detection limit down to 7.62 ng/mL.

## 4. Colorimetric and Naked-Eye Sensors for POPs

**Figure 9:**
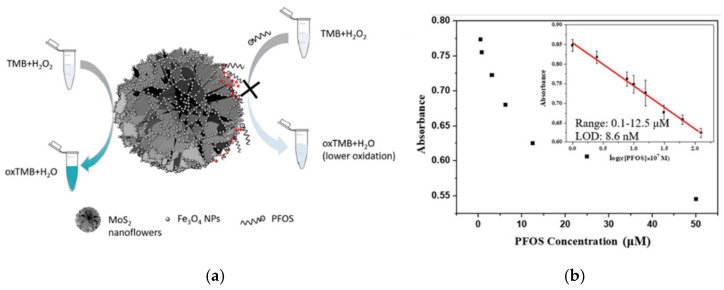
(**a**) Colorimetric sensing detection of PFOS; (**b**) UV–Vis absorbance variation at 652 nm with increasing concentrations of PFOS. Reported from [36].

Colorimetric sensors are also largely exploitable for the rapid and easy monitoring of POPs in the environment by in situ analysis, due to the ease of fabrication, fast response time, high sensitivity, portability, and low production costs [35]. Additionally, neither a highly qualified operator nor the use of complex instruments is necessary to carry out measurements. Rather, frequently familiar and daily devices (smartphones, webcams, laptops, monitors) are used, or even the signal is detectable by naked eye. In the current scenario of colorimetric sensors for the detection of POPs, in the last five years, they have been used for different manufacturing strategies, which involve the exploitation of multiple solid support, as nanomaterials or inorganic compounds, receptors of different nature and several strategies for the conversion of the optical signal. Regarding the use of nanomaterials as solid support, Liu et al. prepared a MoS_2_/Fe_3_O_4_ nanocomposite with 3D structure that shown catalytic activity toward the chromogenic receptor 3,3,5,5-tetramethylbenzidine (TMB), generating a typical blue color when H_2_O_2_ is present [36]. Fe_3_O_4_ nanoparticles, placed mainly along the petals of the 3D structure of MoS_2_, improved dramatically the catalytic activity of the MoS_2_ for TMB oxidation. The reaction provides hydrogen for the reduction of H_2_O_2_ to H_2_O, leading to a clear blue color appearance from colorless solution, as shown in Figure 9a.

In this work, the TMB colorimetric variation toward H_2_O_2_ has been exploited to develop a hypersensitive and low-cost colorimetric sensing system able to detect and quantify perfluorooctane sulfonate (PFOS). Since under weakly acidic conditions the hydroxyl groups of Fe_3_O_4_ nanoparticles are protonated, the PFOS sulfonate groups are attracted by electrostatic interactions and then remain bound by hydrogen bonds. The multiple active sites on nanoparticles surface are thus covered by PFOS, preventing the TMB oxidation catalytic activity of the nanocomposite. The decrease in terms of TMB oxidation will depend on the coverage entity of the catalyst, and therefore on the PFOS concentration. A lower catalytic TMB oxidation leads to a decrease in blue color that is proportional to PFOS concentration, generating a colorimetric sensor of “turn-off” type. The calibration made by following the absorbance decrease at 652 nm by increasing PFOS concentration, proved that this colorimetric approach has a great sensitivity with a good linear correlation in the concentration range [PFOS] = 0.1–12.5 µM, as shown in Figure 9b. However, the system shows some problems with selectivity, being that other compounds able to interact with NPs hydroxyl groups can compete with PFOS, contributing to the lowering of Abs 652 nm and altering the response. This type of analysis uses complex tools for optical signal processing and requires professional laboratories. 

**Figure 10:**
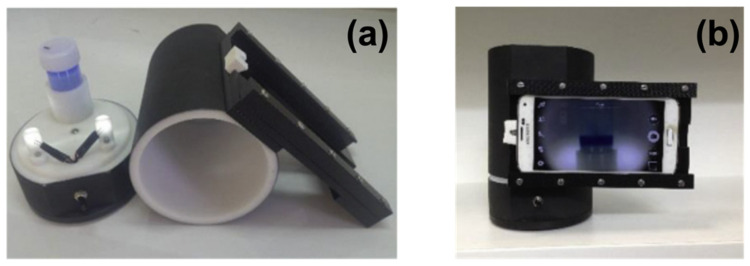
(**a**) The portable instrument setup, with the sample holder and the fixed LEDs. (**b**) The holder keeping the smartphone in a reading position. Reported from [37].

It is well known that the simplest method to detect the presence of a determinate analyte is colorimetric analysis by the naked eye, as for the strips for pH control. In [37], Cheng et al. developed a sensor strategy for the detection of PFOA and PFOS using the operating principle of commercial reading kits, employing a specially developed smartphone app for the optical signal processing in the RGB channels. The colorimetric sensor has been developed using the asktCARE^TM^ kit containing ethyl acetate, the organic solvent for analyte extraction, and the cationic dye ethyl violet. PFOA and PFOS, as surfactants, can electrostatically interact with cationic dye leading to the formation of an ion-pair. The ion pair, having the hydrophilic terminals blocked, is a hydrophobic unit immiscible with the aqueous phase. After mixing the asktCARE^TM^ reagent with the aqueous sample containing PFOS/PFOA, an upper layer of organic phase is formed containing the analyte–dye ion pair. This phase can be extracted, and its optical signal recorded in the RGB channels by the smartphone app; the intensity recorded in the blue channel resulted proportionally to the amount of PFOS or PFOA contained in the original sample. The quantification was possible after calibration with a well-known reference. The performance of the method is guaranteed by the development of a specific instrument setup; this includes a holder to keep the smartphone at the same positions and the use of fixed LED to have a constant lighting independent from environmental conditions, as shown in Figure 10. The sensor was able to detect PFOA and PFOS concentrations as low as 0.5 ppb, but to have such low limits, it is necessary to treat the sample with complicated pre-concentration operations, through solids phase and double liquid phase extraction. Furthermore, this method allows one to perform quick in situ analysis, using as technology, a familiar smartphone and a highly available app.

**Figure 11:**
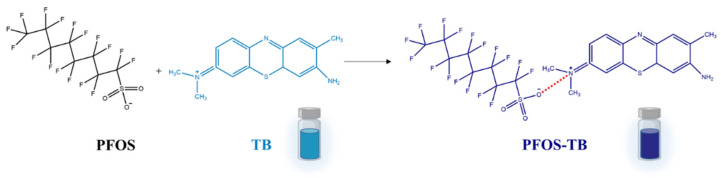
Illustration of the sensing mechanism for PFOS detection based on a TB receptor.

Recently, He et al. [38] reported a detection method for PFOS where any kit or solid support was used for the receptor deposition. The analytical approach consisted simply in the physical mixing of two solutions: the toluidine blue TB receptor and the PFOS sample, avoiding the complex preparation of nanoparticles or other nanomaterials as solid support. TB is a commercial dye, generally used as reagent for RRS (resonance Rayleigh scattering) and colorimetric applications due to its excellent absorption capacity in the visible region. The sensing mechanism that allows for the detection of PFOS is simple; in a weakly acidic environment (pH = 4.0), the TB is found in its monomeric basic form with a positively charged quaternary ammonium group; thus, the anionic PFOS when mixed with TB interacts electrostatically, forming a larger PFOS–TB complex, as shown in Figure 11. The formation of this complex leads to the variation of two optical signals, both depending on the quantity of the analyte PFOS and is therefore useful for sensory purposes: the increase in the intensity of the RRS and the variation of the peak ratios in terms of UV–Vis absorbance. The formation of the TB–PFOS complex results in a decrease in the peak at 602 nm and a growth of a new peak at 502 nm proportional to the increase amount of PFOS. This spectroscopic phenomenon generates a clear colorimetric variation, from a bright blue to a dark blue characteristic of TB and the TB-PFOS complex, respectively. UV–Vis absorption calibration tests showed that this approach provided a LOD value (4.2 mmol/L) comparable to other PFOS detection methods. Besides, this colorimetric approach presents the advantage of being a simple and practical method since the mixing of TB with the sample is only needed, avoiding tedious preparations of nanoparticles or extraction and pre-concentration operation. 

In [39], Taylor and collaborators reported a colorimetric sensor combining the simplicity of using a portable kit, the revelation through the naked eye and the absence of a pre-treatment of the sample to analyze. A porphyrins receptor is synthesized, functionalized in peripheral positions with long fluorinated chains in order to create a kind of cage able to interact with PFOA. Long perfluorated chains interact with the PFOA analyte through two concurrent effects: the multiple C–F–F–C interactions between analyte and peripheral fluorinated receptor chains, and the hydrogen bond between carboxylate and amide functionalities. The host–guest complex leads to a spectroscopic variation that generates a rapid colorimetric variation from red to green. This has been exploited for the detection of PFOA for real aqueous samples: the receptor solubilized in a small volume of dichloromethane is mixed with large-volume aqueous samples leading to a biphasic solution. PFOA is captured by the receptor, which is preconcentrated in the organic phase. Due to the pre-concentration operation, the color change allows one to detect the presence of PFOA up to a detection limit of 3 ppm. The colorimetric sensor enabled the detection of PFOA directly on field and without qualified personnel. However, the method does not allow for the accurate PFOA quantification, but only its presence in the sample analyzed.

## 5. SERS-Based Optical Sensors for POPs

The discovery of the inelastic Raman scattering of photons by molecules happened in 1928 [40], but only fifty years later, this phenomenon proved useful for applicative purposes due to Fleischman and coworkers [41], who reported the first example of amplification of the weak Raman signal. In particular, the first use of Surface Enhanced Raman Spectroscopy (SERS) in sensing was reported in 1999 by Kronfeldt et al., where a silver substrate was exploited to monitor PAHs in seawater with a limit of detection up to nanomolar concentration [42]. Typically, the enhancement of a Raman signal of several orders of magnitude is carried out using metallic substrates that currently are normally into nanostructured materials. The mechanism involved is explained by electromagnetic phenomena [43] occurring at the metal surface (plasmon resonance), which exponentially decays, depending on the distance from the plasmonic hotspots that has to be around 10 nm to observe SERS. In addition, the enhancement through chemical mechanism is possible when the molecule is chemically bonded on the metal surface through covalent bonds, van der Waals or electrostatic interactions, allowing for the occurrence of charge transfer processes between the molecule and the metal surface. 

Detection of POPs exploiting SERS spectroscopy is quite problematic, the main problem concerning the high hydrophobicity of these analytes, which greatly decreases the affinity with the substrate. This results in a lower signal intensity, as it decays exponentially with the distance from the metal surface of the substrate. This role could be best played by SERS-based probes with modified substrates. The use of modified substrates allows one to overcome this obstacle. The functionalization of the metal surface with molecules equipped with functional groups allows for the binding of the analyte and bringing it closer to the substrate area, where the electromagnetic field responsible for SERS enhancement is strong. The adsorption efficiency is driven by the strength of the molecular interaction, and for these reasons, the chemical linker is normally endowed with carboxyl, amine, hydroxyl, or thiol groups able to form covalent, ionic or hydrophobic interactions.

The main functional groups used include cyclodextrins [44], viologen derivatives (such as lucigenin [45] and paraquat [46]), dicarbamates [47], calixarenes [48], humic acids [49], monoclonal antibodies [50], graphene [51] and molecules containing thiol groups [52].

While being quite effective in increasing the LOD and Raman signal, this method tends to distort the specific spectra of each analyte, making the recognition of similar species more difficult. One of the strengths of SERS are spectra, which act as fingerprints for each molecule.

To overcome this problem, substrates with specific geometries, such as gels, foams, nanotubes and nanoparticles, can be used. Among these, the use of metal nanoparticles in combination with molecules suitable for the binding of the analyte has proved to be advantageous for these types of analyzes, considering the ease of preparation and the effects on the increase in the signal, enabling the detection of analytes with a LOD up to 10^−9^–10^−10^ M, even reaching in certain cases the detection of a single molecule [53].

SERS substrates that are normally used include Ag, Au, Cu or Al shaped in a numerous nanostructure and assembled to form solid aggregates, liquid-phase metallic colloids or surfaces fabricated by using different techniques [54]. The most used metals are undoubtedly silver and gold. In particular, NPs that use silver produce a high enhancement of Raman effect. Different types of mixed silver NPs have been prepared, such as mixed silver and cyclodextrin nanoparticles (β-CD-Ag NPs) [55], Fe_3_O_4_ (Fe_3_O_4_@Ag) [56], humic acids (HAs-Ag NPs) [49], PVP (AgNO_3_-PVP dendrites) [57].

Alvarez-Puebla and colleagues [58] have designed and characterized a SERS substrate based on a microporous silica capsule that encapsulated gold plasmonic films. These molecular sieves showed a unique colloidal stability even at high salt concentrations and were tested to scavenging DDT (dichlorodiphenyl–trichloroethane) from natural water sample. Quantitative information on DDT amount were extracted from SERS spectra with a linear correlation from 1 ppb to 3 ppm. Cai et al. in their study [59] adopted a cost effective and sensitive approach by deposition of Ag nanoparticles on a non-woven fabric able to detect by SERS several pesticides residues on fruit samples by rubbing the surface. Low-cost pesticides analysis was reported in the work of Chi et al. [60] where they prepared reusable nanoporous silver (NPAg) sheets with high surface area for SERS detection of organochlorine. SERS signal of lindane was identified and quantified with a limit of detection of 87 ppb. The reproducibility of this SERS analysis was observed even when NPAg sheets were reused 20 times. Lindane was also the target analyte in the work of Zhou et al. [61] where Au concave trioctahedral and calyptriform nanocrystals films were fabricated with a simple low temperature process and showed an enhancement factor higher than 107 with this organochlorine. The intensity of the Raman peak with lindane was correlated with a linear double logarithmic relation from 30 ppb to 300 ppm.

Besides noble metals, even semiconductors manifest surface plasmon resonance effect and can be potentially used as SERS substrates. However, these materials can be decomposed, oxidized or unstable by laser irradiation and therefore not suitable for SERS applications. An exception of this context is the work of Xi at al. [62] where they reported how metallic MoO_2_ nanodumbells can be applied as SERS substrate to detect low concentrations (till 10^−7^ M) of POPs as BPA, DCP, TCP etc.

Eremina et al. prepared a dual-purpose SERS sensor able to detect and quantify simultaneously PAHs and PASHs up to 10 nM concentration [63]. The researchers increased the sensitivity trapping the target pollutants (π-donor) with π -acceptor molecules on the surface of AgNPs. Several π-acceptor molecules were tested and studied even through DFT calculations; DDQ was chosen due to the strongest charge-transfer complexes (CTCs) interactions with the selected pollutants. The developed method was tested in the analysis of real diesel fuel sample where it was found that prior LLE of PAH in the form of the electron donor–acceptor complexes with suitable π-acceptor can increase sensitivity of PASH determination by almost two-fold.

Zhu et al. have developed an innovative system based on Ag-nanocubes/graphene-oxide/Au-nanoparticles composite film on a hydrophobic surface to realize sensitive SERS detection of pesticides [64]. The substrate was built to achieve both high density of hotspots and effective concentrations of the target analyte in water, leading to high SERS activity and detection sensitivity. Aqueous droplets containing the analyte were formed on the hydrophobic surface, and the molecule were concentrated on the SERS active sites due to the hydrophilicity of the GO sheets during evaporation process. This SERS platform was tested in the detection of thiram and thiabendazole in drinking water with LOD of 0.37 ppb and 8.3 ppb, respectively.

The detection and quantitative analysis of dibenzothiophene and its derivatives with a multi-layer Ag NPs modified glass fiber paper as SERS substrate was reported in [65]. The novelty of the approach consisted in detecting DBT without the need of a π-acceptor compound to assist the SERS signal due to the improvement of chemical enhancement induced by multilayer nanoparticles. The researchers found a linear DBT response in concentration range between 10^−3^ and 10^−5^ M with a LOD of 10^−6^ M. Spiked petrol samples were also tested, and the recovery rates calculated were comparable with the ones reported in the literature. 

All the above reported studies are focused on modulating the SERS surface ability to enhance the Raman signal (e.g., generating more hot spots); however, this approach could not be enough to achieve sub ppm analysis of POPs. In order to overcome the adsorption issues and the chemical incompatibility between organochlorine pesticides molecules and noble metal nanoparticles, Zhang et al. used a “bridge” organic substance (diquat, lucigenin) coated over the metal Ag nanoparticles [66]. The droplet concentration method was used to increase analyte Raman signal; the system was characterized by a dual affinity substance toward metal and pesticides, and SERS spectra were due to energy transfer and a molecular resonance mechanism upon laser excitation. The DFT calculated binding energies have shown diquat to interact with the majority of the analytes, while lucinogen was more selective for some organochlorine molecules (op’-DDT, aldrin). 

Another example of bifunctional linker, 4-mercaptophenylboronic acid (4-MBPA) was used by Zhou et al. to connect the surface of Au nanosheets-built hollow sub-microcubes (ANHCs) with the pesticide hexachlorocyclohexane (HCH) [67]. The porous Au cubes, with high density tips and sheets, performs strong SERS effect due to the intense local field enhancement generated at the edges and gaps over the packed nanosheets under laser excitation. HCH was trapped by a Suzuki cross coupling reaction between halogenated (Cl, Br, I) hydrocarbons and the organic boronic acid linker under pH = 7 and 80 °C temperature condition. The researchers were also able to prove differentiations between HCH isomers based on the different SERS spectra obtained.

Ma et al. prepared a new SERS substrate based on gold nanoparticles (AuNPs) due to their better stability and biocompatibility compared to the silver NPs, which instead are characterized by superior plasmonic performances [68]. AuNPs were functionalized with cysteamine for PCP detection, exploiting the electrostatic interaction on ions pairing between the negative charge of PCP in water solution and positive ammonium cysteamine group. The reproducibility and the stability of SERS substrate was improved immobilizing AuNPs on glass surface through gold–thiol covalent bonding. The researchers optimized distribution, uniformity, and size of AuNPs on glass surface with the result to achieve good reproducibility, stability, and reusability to detect PCP in ultrapure and tap water with a LOD of 0.26 μg/L. In addition, the developed SERS system was PCP selective in multicomponent solution containing PCP analogs. 

Meso-AuNPs functionalized with mono-6-thio-β-cyclodextrin (HS-β-CD) were prepared by Zhang et al. to efficiently adsorb PAH (anthracene, naphthalene) via host–guest interaction [69]. The LOD of the two compounds were found below 1 and 10 ppb, respectively. This system can deliver a large surface area and provides an enrichment of analytes concentration due to the high dentistry mesopores and the hydrophobic slippery surface generated by HS-β-CD functionalization. 

**Figure 12:**
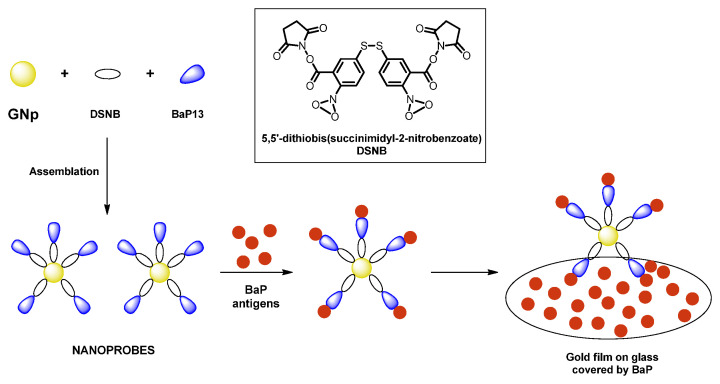
Schematic procedure of sensor assembly and BaP detection. Adopted from [50].

The nanoprobes made by AuNPs labeled with DSNB (5,5′-dithiobis(succinimidyl-2-nitrobenzoate) and functionalized with anti BaP, a monoclonal antibody, have been prepared in [50]. The first one has the dual task of increasing the Raman signal of the analyte and acting as a coordination bridge between NP and antibody, the second guarantees an excellent affinity with the analyte, allowing for the detection of BaP at the nanomolar level (2 nmol/L), as shown in Figure 12.

In [70], excellent results were achieved using sensors mounted on a portable Raman spectrometer having unfunctionalized gold substrates, based on AuNPs colloids in the presence of Cl^−^ ions (from 1 M NaCl) and trisodium citrate. This technique allowed for the detection of numerous PAHs with high LODs, leading to the determination of PYR, PHE and NaP with a LOD of 0.45, 0.23 and 1.38 µg/L, respectively. 

The use of SERS for the recognition of food contaminants would be of great use. As with environmental pollutants, this technique could be used on site to monitor raw meat, fish, mollusks and crustaceans, vegetables and fruit, milk and industrially processed food products quickly and effectively.

**Figure 13:**
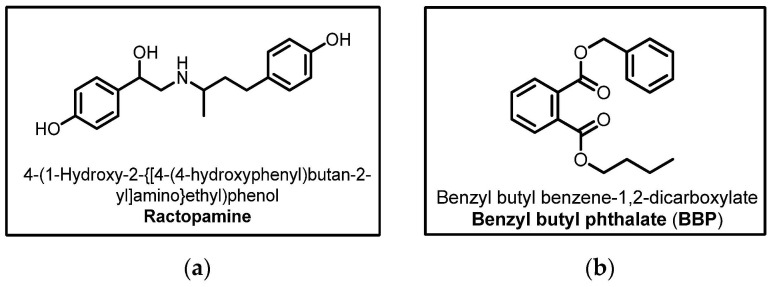
(**a**) Molecular structure of 4-(1-Hydroxy-2-((4-(4-hydroxyphenyl)butan-2-yl)amino) ethyl)phenol (Ractopamine). (**b**) Molecular structure of Benzyl butyl benzene-1,2-dicarboxylate (BBP).

For instance, ractopamine, a β-adrenergic agonist, is currently widely used in pig farms, as shown in Figure 13a. It produces an increase in lean mass in pigs, and its residues in meat can be potentially hazardous to human health. To carry out an effective and rapid detection of ractopamine, SERS substrates based on AgNPs arrays fixed on Al_2_O_3_ nanotips have been developed in [71]. These substrates could be used to probe residual ractopamine directly on raw pork without any pretreatment. Furthermore, the achieved LOD was less than 10^−8^ M, which is lower than the standard international concentration value of 10 µg/L. 

A further example consists in the use of Ag@Fe_3_O_4_@Ag/β-cyclodextrin (CD) nanoparticles in the detection of butyl benzyl phthalates (BBP), as shown in Figure 13b. These compounds are illegally added to liqueurs to increase their viscosity, at the expense of their toxicity. The detecting method consists in the use of a β-CD-functionalized substrate with Ag and Fe_3_O_4_, equipped with hydrophobic inner structures capable of trapping the BBP molecules and generating a complex. The presence of Fe_3_O_4_ allows for a rapid aggregation in nanoparticles under the influence of a magnetic field, accelerating sample processing and increasing sensitivity [72].

The concentration of the analytes on SERS substrate to observe a Raman signal is critical for many POPs, and for example, not all pesticides exhibit equal SERS activity on the same surface. Moreover, real samples contaminated by POPs, such as plants, vegetables, or animal source food may not have a homogenous distribution of chemicals in their bulk or surface, and for this reason, sample preparation before SERS analysis is normally required to validate the technique. Qu and He have achieved an amplification of Raman signal for chlordane that have weak SERS activity on citrate-coated AuNPs [73]. The method consisted of applying a simple rolling protocol on parafilm layering droplets of chlordane methanolic solution with AuNPs to improve the interaction between the analyte and the metal. A prediction model to estimate the amount of chlordane in a complex crude oil based on the experimental Raman intensities and the number of rolling steps, achieving satisfactory recovery values, was established. The developed approach represents a possibility to enrich the concentration of hard-to-detect analytes without the use of other costly instruments.

Solid–liquid/liquid–liquid extraction (SLE/LLE) is another technique that can be necessary for sample preparation and allows for POPs preconcentration with the increase in SERS analysis sensitivity. Zhou et al. exploited the simplicity of LLE step to improve SERS analysis of several PAH in water [74]. LLE was able to increase sensitivity until 2–3 orders the magnitude compared with detection without LLE. The lowest detectable concentrations were 5, 50 and 100 ng/L for pyrene, benzo[a]pyrene and anthracene, respectively. The developed LLE–SERS method was also tested with success in the analysis of real water samples from oceans and costs being spiked by those PAH contaminants. The characteristic Raman peaks of pyrene, benzo[a]pyrene and anthracene were observed together with other peaks coming from the other organic species presents in the water ecosystem.

The exploitation of metal–organic frameworks (MOFs) showed another efficient strategy for SERS detection of POPs. MOFs are composed by entanglement of inorganic nodes (single ions or clusters of ions) coordinated with organic linkers. The well-defined 3D structures are characterized by crystallinity and high porosity as well as high specific surface area. One of the main MOFs advantages is the possibility to adopt rational design approach making versatile structures in terms of properties and affinity toward the binding molecules. Cai and colleagues have designed and prepared an urchin-like Au–Ag alloyed nanocrystal (UANNs) wrapped around a zeolite imidazole framework (ZIF-8) as SERS substrate for the detection of α-HCH and γ-HCH molecules [75]. The thickness of ZIF-8 shell was tuned to find the best SERS performance (20 nm), which allowed HCH to be in proximity with the substrate. The pore size of the MOF has the right dimension to ensure selectivity to the target analytes (<11.6 A). When the HCH concentration is lower than 10^−5^ M, a wrapped enhanced SERS effect due to easier diffusion of the HCH molecules into the pores of the shell layer was observed. ZIF-8 was also the protagonist in the work of Yan et al. where it was successfully employed as a film for Au-Ag/Si nanoporous pillar array to develop a novel SERS substrate for PCP detection [76]. The material was prepared with a layer-by-layer technique having a control over the film thickness and consequently over the SERS effect. The structure of positively charged ZIF-8 could efficiently capture traces of negative charged PCP close to Au-Ag nanoparticles, enabling a large amplification of a SERS signal by surface plasmon resonance effect. This allowed for the achievement a PCP limit of detection of 10^−13^ M and a linear relation with SERS intensity from 10^−7^ M to 10^−13^. 

In [77], Guselnikova et al. used MOF-5 to coat the surface of plasmon–polariton (SPP)-gold grating material for a sensitive, selective of a reproducible detection of organophosphorus pesticides. This work showed how the growth of MOF material over this gold SERS substrate advantageously improves the homogeneity of the samples because it minimizes the metal–metal aggregation phenomena. The developed sensing system achieved a limit of detection lower than 10^−12^ M for paraoxon and fenitrothion with a high Raman signals reproducibility. Moreover, the researcher demonstrated, using extraction procedures with common solvents, that it was possible to use this substrate to successfully analyze mixture with more contaminants in simulated soil samples.

## 6. Optical Biosensors for POPs

Biosensors employ some biochemically mediated recognition, which is detected by an associated, analytically useful signal. The most common biorecognition elements include enzymes (utilized for direct catalytic biosensor and in inhibition-based sensors), antibodies (used in imminosensors), and aptamers, short, single-stranded DNA or RNA molecules that can selectively bind to a target with high affinity and specificity and are used in aptasensors [78]. Despite several drawbacks, for instance a low stability and the necessity of a particular temperature regime for the testing probe preservation prior to use, the development of biosensors for detection of organic pollutants has received considerable attention in recent years due to the possibility to have a miniaturized and portable system to monitor on-site complex matrices [79]. 

**Figure 14:**
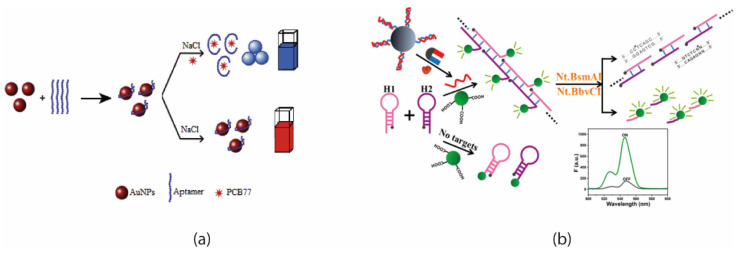
(**a**) Scheme of the colorimetric aptasensor for detection of PCB. The aptamers were absorbed on the nanoparticles. Then, the addition of PCB and the binding with the aptamer induced the aggregation of AuNPS and changed the color of the solution from red to blue. (**b**) Representation of the fluorescent aptasensor for PCB detection with the development of a dual amplification strategy [83].

In this context, Cheng et al. [80] developed a colorimetric aptasensor for the detection of PCB 77. PCBS are colorless compounds that are accumulated in fats, and their levels increase in the upper food chains [81]. They have been classified as carcinogens by the International Agency for Research on Cancer (IARC) [82]. For detection, the authors used AuNPs due to their unique optical properties, high surface area, simple synthesis protocols, high chemical stability and biocompatibility. The addition of PCB 77 to AuNPs protect the nanoparticles from aggregating in the presence of high salt concentration (NaCl). In case of unprotected AuNPs, salt induces aggregation and, as a consequence, a changing in color from red to blue (Figure 14a). As well, the interaction with PCBs produces a change in the structure of aptamers, switching from random coil to advanced structure, which in turn does not protect AuNPs from aggregation and results in a purple–blue color. The minimum detection limit was 0.05 nM, and the system was found to be selective versus interferents with other PCBs. The concentrations provided by the proposed method were in great accordance with those obtained by means of the analytical techniques such as gas chromatography and mass spectrometry. Wang et al. [83] introduced the use of upconversion nanoparticles (UCNPs), a new class of imaging agents that showed long lifetime, absence of autofluorescence, high photo-stability and low toxicity. The principle of the fluorescent aptasensor for PCB was based on the dual-amplification strategy using nicking endonuclease and hybridization chain reaction (HCR). The whole sensing mechanism is schematized in Figure 14b. Two harpins (H1 and H2) were first designed according to the partial complementary sequence of cDNA of PCB72/106. The aptamers/cDNA complex and magnetic microspheres (MMPs) were linked together. In the absence of PCB72/106, the HCR process cannot be triggered, and the UCNPs were connected to the hairpin. In this case, quenching occurred since the close distance between the quencher BHQ-1 and UCNPs units induces a fluorescence resonance energy transfer (FRET) mechanism. Conversely, when the target was present, the aptamers recognized and bound to the target and the cDNA was released from the microspheres. The released cDNA could initiate HCR and open the stems of H1 and H2 that formed a long double-stranded DNA. The UCNPs connected to the open hairpin structure, and fluorescence was recovered since the distance between the UCNPs and BHQ-1 was further away. In this first amplification step, the UCNPs and BHQ-1 units are still relatively close each other. A second amplification mechanism was introduced by nicking endonuclease that selectively cut the double-stranded DNA to remove UCNPs. These latter units were then definitely moved away from BHQ1 unit producing a complete fluorescence recovery. The proposed system allowed for the detection of PCB72/106 with 0.0035 ng/mL as the detection limit.

**Figure 15:**
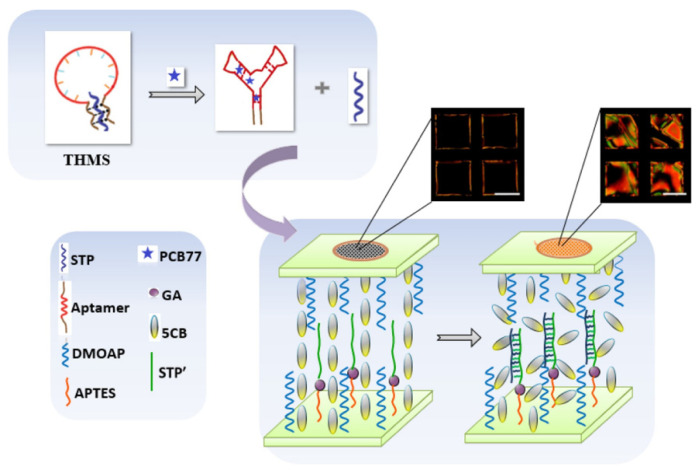
The schematic structure of an aptamer-based LC biosensor for PCB. After the binding with the target, the structure of THMS is disassembled, and the STP binds STP’ on the LP cell with a change of optical response, from dark to bright. Reported from [84].

Verdian et al. [84] developed a novel liquid crystal (LC)-based aptamer biosensor to detect PCB77 with the use of a triple-helix molecular switch (THMS) structure [85,86,87]. They used the THMS because its high stability, selectivity, and affinity of the original aptamer, as shown in Figure 15, which illustrates the LC aptasensor for PCB77 based on the THMS structure. The aptamer is based on triple-helix molecular switch (THMS) formed by pre-hybridization of a central, target-specific aptamer sequence (red) flanked by two terminal fragments (brown) with another fragment STP (in navy blue). When the pollutant is present, the aptamer binds to the target that disrupts the arrangement of the THMS and STP binds with STP’ on LC cell. This bind involved a change of optical response from dark to bright in the range of 1.5 × 10^−5^ to 15 μg/L, obtaining a lower detection limit for PCB77, 1.5 × 10^−5^ μg/L. The sensor was tested on real samples as water and milk. The obtained results showed that this LC aptasensor was reliable for food safety and environmental analysis, without the use of a sophisticated and expensive device in the detection. 

Polybrominated diphenyl ethers (PBDEs) are a class of synthetic halogenated organic compounds used as flame retardants. PBDES is hydrophobic and presents a high resistance to biodegradation. The main challenge to the develop of a PBDE-detecting biosensors is the lack of a specific receptor protein. Whole-cell biosensors are actively utilized as biorecognition elements. To overcome this drawback, Chen et al. proposed the use of compounds with high cell surface hydrophobicity (CSH) [88]. Particularly, the authors have utilized a novel bacteria strain, *Sphingobium xenophagum*, which was found in the river sediment of a high polluted area in Guiyu, China [89]. A genetically engineered CSH cell, *Sphingobium xenophagum C1*, expressing firefly luciferase in place of Chr1_2466 on the cell membrane served as a whole-cell biosensor for PBDEs detecting. From experiments, C1 resulted as highly specific for PBDEs with minimal interference from compounds having structural analogies. The extracellular luminescence intensity of the C1 biosensor showed a linear range from 0.05 to 6.0 µM of deca-BDE with a detection limit of 0.01 µM. Compared to the hydrophilic strain as a chassis cell (C2), C1 biosensor significantly increased the bioavailability and sensitivity of PBDEs.

**Figure 16:**
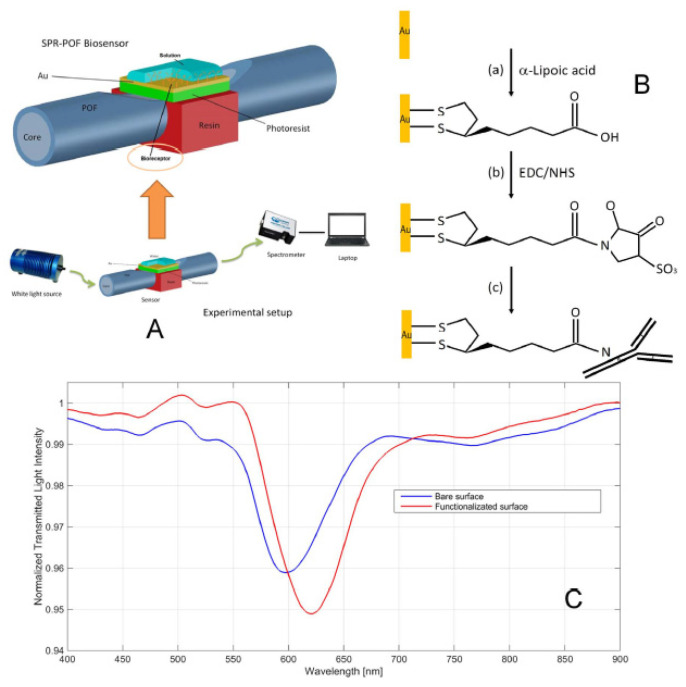
(**A**) Optical biosensor system based on the POF–SPR platform. (**B**) Functionalization process of the gold surface. (**C**) Resonance spectra acquired in buffer solution obtained before and after the functionalization process with msAb anti-PFOA. Both measurements were obtained by dropping 50 μL of 20 sodium phosphate buffer pH 7.4 over the sensing surface, with and without the receptor layer. Reprinted from [92].

Another class of pollutants largely utilized in industrial and commercial products is perfluorinated compounds (PFCs) [90,91]. Cennamo et al. [92] developed and characterized a new surface plasmon resonance optical fiber (SPR–POF, see Section 7.2 for more details) biosensor to detect PFOA/PFOS, as shown in Figure 16A. To allow for binding with the PFOA, the gold surface of the SPR–POF chip was treated with a solution of α-lipoic acid (a), EDC/NHS (b) and finally with mono-specific antibodies previously designed to be selective toward PFOA/PFOS (c), as shown in Figure 16B. The binding of antibodies on the sensor surface is confirmed by the SPR transmission curve obtained before and after the functionalization, as shown in Figure 16C. This shift of resonance wavelength indicates that the refractive index in contact with the gold surface is increased, showing the presence of antibodies on the gold surface. This system LOD was 0.21 ppb, which is lower than the maximum residue limit of PFOA, fixed at 0.5 ppb by European Union regulations. The SPR curves with different concentrations of PFOA (0–100 ppb) were registered to study the non-specific binding between the gold surface and analyte. 

## 7. Other Types of Optical Sensors for POPs

### 7.1. Photonic Crystal Sensors

The application of photonic crystals (PhC) for the development of chemical optical sensors is based on their optical properties variation upon the influence of analytes, resulting in visually observable changes under optimum illumination [93]. PhC normally consists of periodically arranged dielectric materials having different refractive indexes and producing photonic stopband and color when the stopband is located in visible range [94]. The sensing technique is based on the measuring the PhC transmission/reflection magnitude variation upon the interaction between the light and the analyte. The measured variables are most often the wavelength of peak reflectance and/or the value of peak reflectance, either as a function of space or as a function of time. Since the PhC sensor responses are reliable for small areas (as small as a few μm^2^ to mm^2^), these sensors can be implemented, for instance in Lab-on-Chip devices for in situ sensing of the wide range of analytes. 

Recently, the PhCs sensors for small aromatic organic molecules and POPs detection were reported. Kou et al. have developed the nanoporous multilayered organic–inorganic composite PC sensors prepared through alternate assembly of poly(styrene-acrylic acid) and TiO_2_ nanoparticles for a visual naked-eye detection of hazardous volatile aromatic hydrocarbon vapors. The sensor was tested for different concentrations of benzene, toluene, xylene and 1,2,4-trimethylbenzene (TMB) vapors with LODs for the last two tested compounds of 99.2 ppm (for xylene) and 14.7 ppm (for 1,2,4-TMB) [95]. The colorimetric analysis program on a smartphone was developed for toluene assessment as an example, and through color captured with the program, the analyte concentration values were easily obtained, thus demonstrating the potential of PhCs devices use in high-performance aromatic VOCs optical sensors for environment quality monitoring.

In [96], the possibility to detect water impurities, and among them DDT (dichloride diphenyl trichloroethane) and PCB (polychlorinated biphenyls) through measuring their refractive index (RI) values by using a hexagonal–structured photonic crystal (PhC) optical sensor designed using the OptiFDTD program code was investigated. By passing the optical light with pulse center frequency of 0.15 and pulse width of 0.1 through the proposed PhC structure (square lattice with rods radius of 0.19 μm in air configuration, the lattice constant “a” = 1 μm, Silicon slab dielectric constant e = 12), the amplitude and wavelength shifts were observed by changing RI as per the impurities that may present in drinking water: DDT, PCB, arsenic, mercury, lead, chorine, aluminum, and fluoride, thus indicating the feasibility of the proposed device for future for sensing applications.

### 7.2. Fiber Optic Chemical Sensors (FOCS) and Surface Plasmon Resonance (SPR) Sensors 

**Scheme 4:**
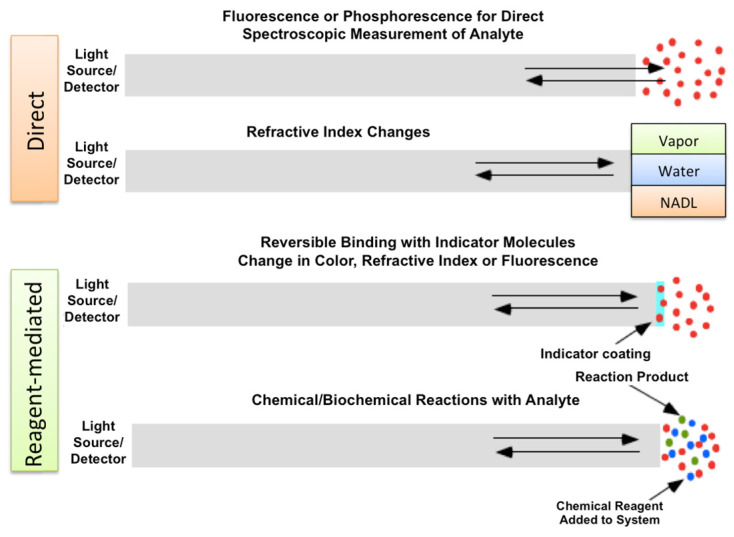
Schematic presentation of direct and reagent mediated FOCSs.

The use of optical fibers has permitted the development of fiber optic chemical sensors (FOCS) based on various sensing phenomena [97] that have found application in many analytical fields [98]. In fiber optic chemical sensors (FOCS), the light is generated by a light source and is sent through an optical fiber. The light then returns through the optical fiber and is captured by a photo detector. Some optical fiber sensors use a single optical fiber, while others use separate optical fibers for the light source and for the detector. In direct FOCSs, the analyte is detected directly via some intrinsic optical property such as, for example, absorption or luminescence. In reagent-mediated sensing systems, a change in the optical response of an intermediate agent, usually an analyte-sensitive dye molecule, is used to monitor analyte concentration, Scheme 4.

The last generation FOCS sensors use the combination of surface plasmon resonance (SPR) sensing platform on plastic optical fibers (POFs), or use photonic crystal fibers (PCF). In addition, micro-structured optical fibers (MOFs), having a certain distribution of air holes in their structure, have been actively used recently for sensing applications for detecting liquids, gases, or volatile organic compounds (VOCs) [99].

SPR is a wave-sensing technique that can detect biochemical reactions in the near-field region. The SPR platform is based on surface plasmon polariton—the electromagnetic waves propagating on the negative permittivity/dielectric material interface boundary that are extremely sensitive to variations on this boundary. The SPR phenomenon was discovered by Wood in the 1900s [100]. Currently, the SPR technique most often uses the reflectivity measurements to detect molecular adsorption of different analytes, such as polymers, proteins, etc., on sensing (metallic or other conducting) surfaces [101].

**Figure 17:**
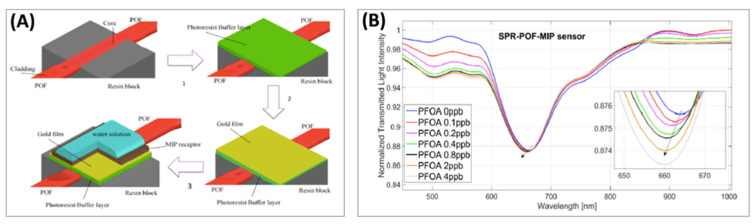
(**A**) Production steps for realizing an SPR sensor in a D-shaped POF with an MIP receptor; 1, 2, 3 correspond to the production steps. (**B**) SPR spectra obtained at different concentrations of PFOA in water solution (0–4 ppb) by an SPR–POF–MIP sensor. Inset: zoom of the resonance wavelengths. Reprinted from [102].

After the development of the SPR biosensor described above [92], Cennamo et al. have reported in [102] the SPR sensor platform using D-shaped POF (980 μm PMMA core with 10 um fluorinated polymer cladding covered with a photoresist buffer layer of 1.5 μm and a thin Au film of 60 nm) modified with a molecularly imprinted polymer (MIP, based on VBT and PFDA functional monomers, EDMA cross-linker and ammonium perfluorooctanoate, FPO-NH_4_ template) receptor for perfluorinated alkylated substances (PFAs) sensing in water, as shown in Figure 17.

The developed SPR–POF–MIP has shown an enhanced response to perfluorooctanoate (PFOA), perfluorooctanesulfonate (PFOS) or PFA contaminants in the C4–C11 range with an LOD down to 0.13–0.15 ppb. The advantage of low cost, good reproducibility and lower LOD of a developed SPR–POF–MIP sensor in comparison to the immunosensor based on a specific PFOA antibody deposited on the same optical and previously reported by the same research group (SPR–POF sensor based on bio-receptors had a LOD of 224 ppt) [92] makes it a particularly promising device for POPs environmental monitoring. Further, the simplified SPR–POF platform combined with the same MIP receptor and employing an experimental setup only based on an LED and two photodetectors. The sensor LOD of 0.5 ppb is compatible with the maximum residue limit fixed by the European Union regulations in the monitoring of PFAs compounds in surface waters [103].

Kaur and Singh have reported the multichannel SPR sensor designed with two concentric channels, with an external coating of gold (Au) on solid silica [104]. The multiple analyte analysis possibility was shown for a developed sensing platform through two different propagating modes operating in the first and second channel. The proposed system has a wavelength sensitivity of 1000 and 3750 nm/RIU (refractive index unit), respectively, for two channels and which is suitable for the analysis of chemical and biochemical analytes, including POPs, with refractive indexes in the range of 1.30–1.40. Recently, Islam et al. have reported the SPR biosensor with hexagonal lattice photonic crystal fiber (PCF) prepared with stable plasmonic material gold (Au) and a thin film of TiO_2_ placed on the glass surface to allure the field from the core guided mode and enhance the adhesion of Au on the fiber [105]. Both x- and y-polarization planes were investigated and provided with high wavelength sensitivity of 16,000 and 17,000 nm/RIU, respectively, with the overall sensing RI range from 1.33 to 1.41, suitable for the detection of unknown biochemical analytes.

## 8. Multisensor Systems for Optical Analysis of POPs

In multisensor analysis, an array of sensing elements produces an individual analyte pattern of responses as a result of a series of low or non-specific interactions. The concomitant responses of several cross-sensitive elements present in the optical sensor array can be processed by chemometric methods for data treatment either to find correlations between sensory array responses and concrete contaminant concentration, or for recognition of different POPs categories and toxicity estimations. Among the chemometric techniques most often applied for POPs optical multisensory analysis, there are principal component analysis (PCA), cluster and hierarchical cluster analysis (CA, HCA), linear discriminant analysis (LDA), and various regression methods. Detailed descriptions of common pattern recognition protocols employed in multivariate analysis can be found elsewhere [8,106,107]^.^

**Figure 18:**
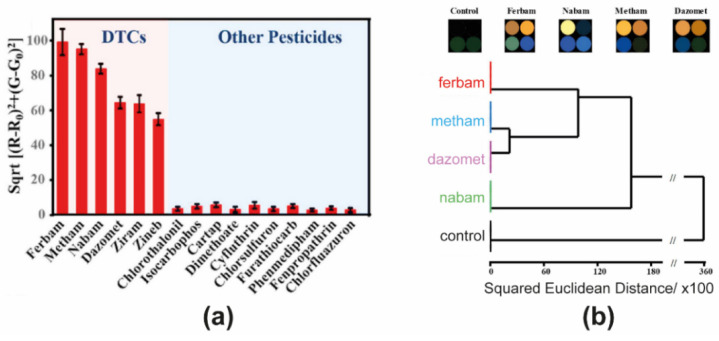
(**a**) Selectivity study of the CuNCs-based sensor. The concentration of DTCs and other pesticides is 50 and 125 mg/kg, respectively. (**b**) The discrimination of four DTCs by using hierarchical clustering. Image is rearranged from Figures 3b and 4 of [108].

Recently, L. Feng et al. proposed a 2 × 2 array based on both colorimetric and fluorometric sensors for the detection of dithiocarbamates (DTCs) using CTAB encapsulated fluorescent copper nanoclusters [108]. DTCs are of great importance in agriculture due to their metal-binding ability, antifungal activity and low acute toxicity associated with low production costs. DTCs are mono-anionic chelating agents due to the presence of sulfur atoms that may participate in the metal coordination. This property can be utilized not only for using DTCs as remediating agents but also as chemical sensing materials to detect heavy metals. In the manuscript, the authors adopted a reverse approach where metal clusters were utilized as sensing substrate for interacting with DTCs. Nanoclusters were produced by a precipitation method utilizing CTAB as a complexant for reduced Cu(I); as a result, low water-soluble micelles were formed because of the long alkane chains borne by the surfactant. TEM micrography revealed an average cluster diameter of around 6 nm. Optically, Cu Nano Clusters (CuNCs) resulted in the emerging of a broad absorbance peak in the near UV region (centered around 300 nm) and the appearance of a PL emission band at 620 nm that the authors tentatively ascribed to the [Cu_4_X_6_]2^−^ structures. CuNCs were then utilized to develop a sensing platform for DTCs by employing micro paper-based analytical device (μPAD) technology where each indicator was immobilized in a central reaction spot of about 5 mm diameter on the paper strip. The sensor array comprised four spots consisting of two colorimetric and two fluorometric sensing units, both obtained from two indicator solutions (25 mg copper cluster/1 mL PVA–PEG solution and 40 mg copper cluster/1 mL PVA–PEG solution) where PVA–PEG were selected as solubilizer and immobilization agents. After having proven the broad pH tolerance of sensing elements, metham sodium was utilized as a representative of DTC derivatives to investigate the sensitivity of an array obtaining good linearity (1–100 mg/L), sensitivity (1.158 mg/L) and limit of detection (0.63 mg/L). The selectivity of a CuNCs-based sensor was further investigated by testing organophosphorus, chlorothalonil, thiocarbamates, carbamates, and pyrethroids. As shown in Figure 18a, only the presence of dithiocarbamates derivatives led to the dramatic fluorescence quenching. XPS investigation provided an insight into sensing mechanisms elucidating that the addition of metham gave rise to a reduction of Cu^2+^ to Cu^+^, resulting in a quenching of fluorescence due to structural changes in the copper halide complex core.

In the case of the array, the multivariate data provided by the 2 × 2 array were further utilized to recognize ferbam, nabam, metham, and dazomet. Samples from these four DTCs were clustered by using Euclidean distance between array responses utilizing hierarchical clustering analysis, as shown in Figure 18b.

**Figure 19:**
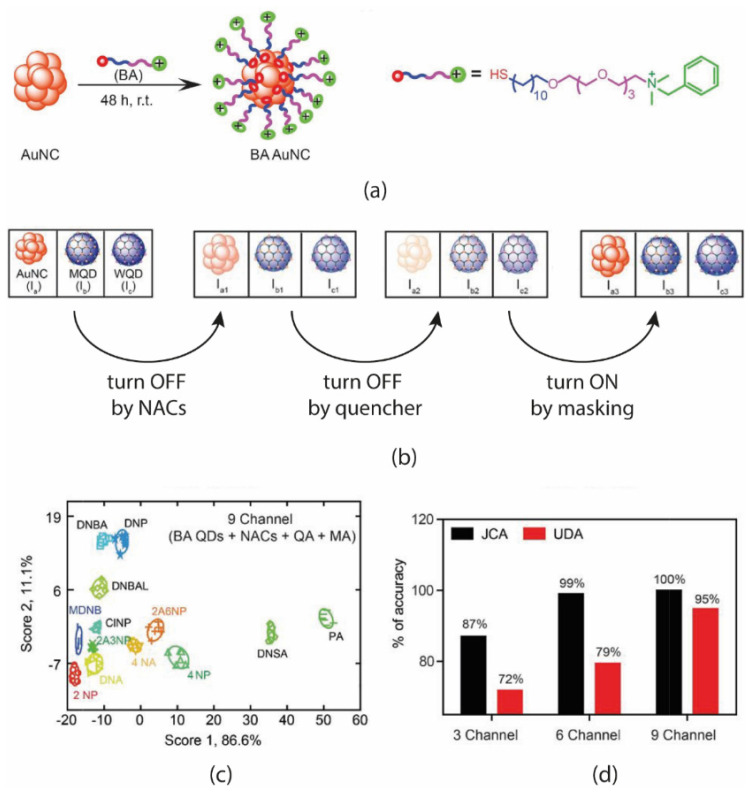
(**a**) Pristine and functionalized QDs. Surface functionalization occurs by the use of thiols bearing a benzene at the antipode. (**b**) Scheme of ON-OFF protocol utilized to improve the array performances. Three consecutive steps were performed consisting of NACs interaction, addition of a quencher (Q.A.) and finally addition of a masking agent (M.A.) to recover the signal of fluorescence. (**c**) PLS-DA projection using nine channels (three sensing QDS × three measurement steps). (**d**) Classification performances after NACs addition (three channels), NACs + Q.A. (six channels), and NACs + Q.A. + M.A. (nine channels). The histograms report both Jackknifed classification accuracy and unknown detection accuracy. Image is rearranged from Figures 1, 5 and 6 of [109].

Concerning the possibility to QDs as sensing elements in multisensorial platforms, De et al. [109] recently investigated the potentialities of functionalized QDs to detect, recognize, and quantify nitroaromatic compounds in aqueous media by fluorescence quenching/recovery protocol. In the paper, the authors did not produce a real sensor array but rather investigated the three fluorophores separately, and in a second stage, they combined the responses to perform multivariate data analysis such as LDA. Initially pristine quantum dots, consisting of gold nanoclusters (AuNCs), MoS_2_ quantum dots (MQD) and WS_2_ quantum dots (WQD) with 3, 3.5 and 2.75 nm average diameters were used as fluorescent receptors. Since usually the main mechanism for the detection of NACs is the electron transfer process between electron-deficient NACs and electron-rich species, the authors utilized both intrinsic electron-rich quantum dots and surface-modified QDs. In this latter case, all the quantum dots were easily functionalized with ligands bearing a thiol termination from one side; whereas the non-thiol end was equipped with an electron-rich benzene group to promote the interaction with the electron deficient NACs, as shown in Figure 19a. 

Furthermore, dense thiol functionalization is supposed to improve the QDs emission by reducing the occurrence of collisions between clusters. It was observed that all the QDs favor the inner-filter effect for quenching the NACs because of overlapping of quencher absorption with either the fluorophore absorption or emission wavelengths. Lifetime investigations showed that AuNCs undergo a dynamic mode of quenching, whereas MQD and WQD favor a static mode of quenching. Likely, the main novelty of this work lies in the protocol adopted for the measurements and the following feature extraction process. The authors utilized what they named sequential ON–OFF strategy to enhance the sensitivity/selectivity of the array. This strategy is briefly reported in Figure 19b and consists of three phase measurements and appears to be an off–off–on approach: (1) the addition of NACs induced a quenching of fluorescence (turn-off by analytes); (2) the addition of quenching agents induced a second stronger reduction of fluorescence signal (turn-off by quencher); (3) the final addition of masking agent re-established the fluorescence intensity (turn-on by masking agents). The optimized array consisted of 2 mg/mL AuNCs, 0.5 mg/mL of MQD and 0.5 mg/mL of WQD. Cu2^+^ (3 mM)/EDTA (4.5 mM) and Fe3^+^ (7.5 mM)/F^−^ (10 mM) were taken as a quencher/masker pair for Au and non-Au quantum dots. Changes in fluorescence intensity were calculated considering the value after and before each or the three additions described in the on–off protocol. Since this protocol produced three “channels” per sensor, up to nine features were extrapolated from the three-element array. After the optimization, 13 NAC analytes (0.1 mM) were tested by the addition of analytes, quenching and masking agents. The resultant LDA score plot (Figure 19c) showed the capability of clustering of all the different analytes further confirmed by the high classification rates obtained (Figure 2d) in the case of both functionalization and strategy.

**Figure 20:**
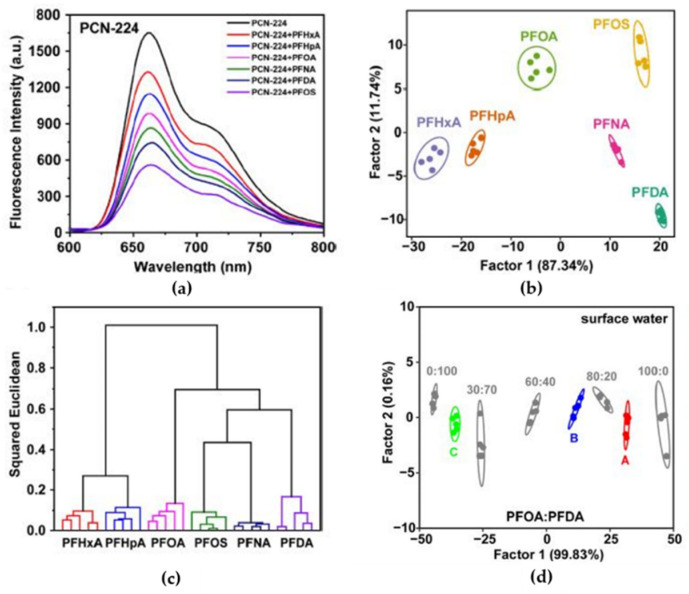
(**a**) Fluorescence spectra of PCN-224 suspension at λ_ex_ 430 nm upon the addition of different PFASs at 2 µg/mL. (**b**) LDA canonical score plot and (**c**) HCA plot derived from fluorescence responses of PCN sensor array toward six PFASs at a concentration of 2 µg/mL. Eclipses represent 95% confidence. (**d**) LDA canonical score plot derived from fluorescence responses of PCN array toward standard (gray) and test (red, blue, and green) mixtures of PFOA and PFDA in surface water. Image is rearranged from Figures 2c, 3b,c and 7a of [110].

Chen et al. developed a sensor array to detect perfluoroalkyl substances (PFASs) in water [110]. The wide use of PFAS in the industrial field results in a large release of these pollutants into the water due to their good solubility, and today, these contaminants represent a threat to humanity due to their toxicity. The idea of the work was to use a sensor array in order to discriminate between different PFAS, allowing for the simultaneous sensing of chemicals with similar structures and properties. Three different luminescent metal–organic frameworks (LMOFs) were produced from zirconium clusters and water-soluble porphyrin, TCPP (tetrakis(4-carboxyphenyl)porphyrin)), and used as sensing materials (PCN-222, PCN-223, PCN-224). When excited with radiation at 430 nm, PCNs showed stable red fluorescent emission, which decreased in the presence of PFAS due to static quenching. For example, Figure 20a shows how the fluorescence emission of PCN-224 was quenched by different PFASs at a fixed concentration. This quenching increased with the concentration of pollutants with a good time constant, around 10 s, due to the nanoporosity of the active material. Since each PCN responded differently to the different PFASs, it was possible to build an array of PCNs able to discriminate PFASs. The sensor array was prepared by pouring PCN solutions (50 μg/mL, in 10 mM NaAc−HAc buffer/pH 5.0) in a 7 × 5 matrix obtained from a well-plate (six PFAS columns plus a column for blank; five replicates in five rows). Figure 20b,c shows the LDA canonical score plot and hierarchical cluster analysis (HCA) related to the responses of the array (namely changes in fluorescence intensity at 430/660 nm in the presence of PFASs compared to that in absence of PFASs) against six selected PFASs, each at 2 µg/mL concentration. Both methods led to the separation of the six analytes into as many groups. Repeating the experiment with lower concentrations of the analytes, the authors established a limit of detection (LOD) around 10^−8^ mol/L. These sensors were able to discriminate PFAS mixtures. Lastly, the sensor array was tested using complex sample matrices—surface water and groundwater—as backgrounds to which PFOA and PFDA were added at three different ratios (A, B and C sample) and compared to standard samples to estimate their concentrations, as shown in Figure 20d. The PCN sensor array was also able to discriminate PFASs in real water samples.

A reusable sensor array capable of quickly detecting the presence of PAHs was reported in [111]. These compounds arise from the incomplete combustion of organic materials, are toxic and remain for long periods in the environment. The sensor array proposed by the authors is based on fluorescence recording, which in turn is modulated by analyte concentrations because of the inner filter effect (IFE). Sensing materials consisted of four quantum carbon dots (CD) coated with polyvinyl alcohol (PVA), which prevented interfering effects between CDs and heavy metal ions without significantly reducing the emission intensities of quantum dots. Being composed of multiple aromatic rings, PAHs show a substantial absorption in the UV and visible regions (specifically between 265 and 420 nm) that overlap with the excitation spectra of the most common fluorophores; this makes them as excellent absorbers for IFE. In the work, the four array elements are excited at five different wavelengths (namely 280, 320, 350, 380 and 410 nm), for a total of 20 channels for fluorescent signals. In addition, there were eight additional channels that directly recorded the absorption of PAHs at eight different wavelengths (namely 280, 300, 320, 340, 360, 380, 400 and 420 nm), for a total of 28 channels. The array was able to discriminate PAHs through the differences in quenching induced by the pollutants themselves in the 28 channels considered. The response of the array was tested against 16 PAHs, prepared at 10 μM concentration, and recorded 15 measures for each. As a result, a 28 × 16 × 15 training matrix was processed through LDA, obtaining a good separation of all the classes in the first two canonical components. Quantification of PAHs is conducted by measuring each of the 16 analytes in the concentration range 0–200 μM. LDA showed that all the compounds’ distributions originated from zero and then gradually diverged in different directions as the concentration increased, without overlapping each other. According to these measurements, the authors built an LDA response library model that allowed one to qualitatively identify PAHs present in a real unknown sample. The lowest detection limit was recorded for benzo[*a*]pyrene (BaP) and was found to be 57 nM. In the case of real samples prepared by adding two anonymous PAHs to the soil and lakebed sludge, the authors were able to determine the concentration of pollutants and to attribute them to the category of the particular PAHs. These results were confirmed by high performance liquid chromatography (HPLC) analysis.

Tropp and coworkers have studied a sensor array for the detection of 12 azo dyes in water [112]. These species contain azo bonds (-N=N-) and are widely used as industrial dyes. Due to their chemical stability, they may remain for long periods in the environment, representing a serious problem for humanity and wildlife. The main problems underlying their detection depend on the great similarity between the different dyes, the complexity of the environment in which they are dispersed (e.g., wastewater) and the low concentrations tolerable by the environment. The idea of this work is to use an IFE-based fluorescence sensing platform. The array contains conjugated polymers (CPs) as active material, while the azo dyes act as an apparent quencher of polymer fluorescence. CPs, due to the extensive delocalized electron systems, give high fluorescence yields. In this regard, the authors prepared three anionic conjugated polyelectrolytes (CPEs), namely copolymers based on fluorene linked to three different comonomers: poly-[2,7-(9,9-bis (4′-sulfonatobutyl)-fluorene)-alt-co-ethynyl] (**P1**), poly-[2,7-(9,9-bis (4′-sulfonatobutyl) fluorene)-alt-co-2,5-thiophene] (**P2**) and poly-[2,7-(9,9-bis (4′-sulfonatobutyl) fluorene)-alt-co-2,2′-bithiophene] (**P3**). The multiple negative charges of CPEs likely limited direct interaction with negatively charged azo dyes, in turn preventing quenching mechanisms other than IFE. The copolymers P1, P2, P3 thus constructed showed an absorption peak at 365, 413 and 454 and an emission peak at 405, 464 and 546, respectively. In this way, it was possible to have a sufficiently extended region that overlaps with that of the absorption of the main azo dyes. In addition to this case, by measuring the intensity of fluorescence in the absence and in the presence of a particular analyte, it was possible to discriminate between different pollutants. The authors prepared the sensor array starting from 10 μM solutions of P1–P3 in H_2_O, combining them with each of the 12 selected azo dyes and placing them in a 384-well microplate. Each of the combinations presented 14 replicates, while the measurements—both in absorption and in fluorescence—were acquired by means of a microwell plate reader. The raw data were processed via LDA. The excellent results obtained confirmed that the introduction of CPs allowed for the discrimination between the different azo dyes in real samples (for example rivers and wastewater) in which the concentration of pollutants was approximately 500 nM. Finally, the sensor array was tested with excellent results toward the discrimination of six azo dyes in a seawater sample in which these analytes were dissolved in a concentration of 15 μM. Although a seawater background may attenuate the fluorescence response due to the salinity and the presence of interfering species, LDA and PCA models were able to efficiently separate the different azo dyes. Moreover, the wide choice in the preparation of the CPs allows for the modulation of the absorption and emission characteristics in order to adapt them to the detection of azo dyes of the required color.

In [113], a sensor array for organophosphorus (OPs) and carbamate pesticides detection was developed. Because of the enormous quantity of pesticides produced and their worldwide diffusion, the development of fast and inexpensive methods becomes crucial, using the so-called QuEChERS (Quick, Easy, Cheap, Effective, Rugged, and Safe) detection method. In this paper, the authors exploited the ability of OPs and carbamate to inhibit cholinesterase (ChE) from producing thiocholines (TCh) by the hydrolysis of butyryl/acetylthiocholine iodide. The presence of these pollutants can be detected by optical sensors that, in turn, measure the fluorescence due to hydrolyzed TCh. In particular, four fluorescent probes with different emission channels and α, β-unsaturated ketone structures were synthesized to detect TCh: N-(9-acridyl)maleimide (**TS-1**), 1-(3-hydroxyphenyl)-3-pyrenyl-2-propene-1-one (**TS-2**), fluorescein-5-maleimide (**TS-3**), diethyl 4-((1H-phenanthro [9,10-d]imidazol-2-yl) phenyl methylene)malonate (**TS-4**). 

**Figure 21:**
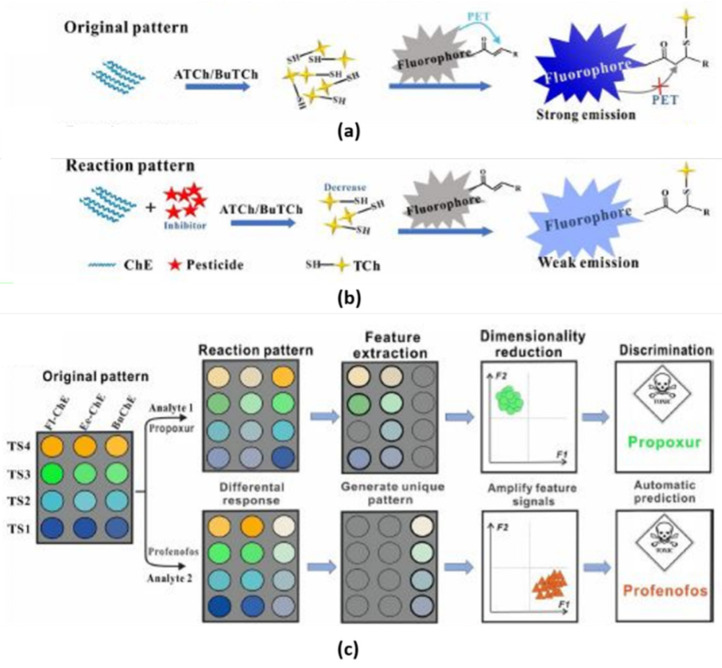
(**a**) The process of discrimination pesticides against OPs profenofos and carbamates propoxur, by using a 12-element array. (**b**) The fluorescence emission mechanism in the original pattern and (**c**) the pesticide induced fluorescence quenching mechanism in the reaction pattern. Image is rearranged from Scheme 1 of [113].

At the same time, three different enzymes were utilized, namely acetylcholinesterase (AChE) extracted from both electric eel (EE-ChE) and fly (Fl-ChE), and butyrylcholinesterase (BuChE) extracted from horse serum. In order to differentiate OPs from each other, the proposed sensing mechanism assumed that different pesticides could inhibit the action of AChE to different degrees. By combining enzymes and TCh sensors, 12 detection channels were obtained. For each of the 12 detection channels, the authors recorded fluorescence response variation at four wavelengths (namely 430, 472, 517, 558 nm), chosen to cover the emission spectrum of the four TS, thus obtaining a 48-channel matrix. In the absence of analyte, ChEs hydrolyzed AtCh/BuTCh, producing TCh with a consequent increase in fluorescence signal. The recovery of fluorescence is due to a reaction between sulfydryl and unsaturated ketone, which leads to the destruction of the unsaturated structure in the recognition site of TSs, removing the fluorescence inhibition due to the photoinduced electron transfer (PET) process, as shown in Figure 21a. Conversely, the presence of pesticides led to inhibition of the three ChEs and therefore to less TCh produced, which in turn resulted in a lower fluorescence yield, as shown in Figure 21b. The different affinity of TCh with the four TS combined with the different degrees of inhibition of the pesticides toward the three ChEs made discrimination possible, as shown in Figure 21c. In the manuscript, this data collection system combined with LDA processing allowed for the discrimination between 30 OPs and carbamate up to concentrations equal to 0.2 ppm. Moreover, the data collected made it possible to create a database for the recognition of real unknown samples.

## 9. Conclusions and Future Perspectives

Chemical sensors are of great interest for many high social and economic impact fields, such as environmental monitoring, processing control, food analysis and medical diagnostic. The rising levels of POPs pollution due to an expanding economy and the ongoing tightening of air and water quality standards have supported the continuous demand for novel chemical sensors. The sensor market over the last decade is experiencing a constant increment with nearly five percent increase per year. In 2020, it was valued at USD 21.39 billion, and is expected to reach a value of USD 32.96 billion by 2026, with growth of 7.51% [114]. Among the chemical sensors with different transduction principles, optical sensors are of particular interest and attention, due to their simplicity in preparation and signal acquisition, low costs, and fast response time [114]. The critical point to a wide exploitation of optical chemical sensors is related to the development of reliable devices, able to satisfy the requirements of target applications. The standard laboratory sensory techniques have become less requested due to the transferring sensory technologies toward the everyday application by non-experienced wide-range users and simplification of sensors construction, lowering the costs and simplifying (or avoiding at all) the equipment involved.

In this paper, the recent developments in innovative optical sensing systems for the effective monitoring of and health risk assessment related to the persistent organic pollutants in the environment were reviewed. The optical chemical sensors and biosensors with different operating mechanisms, employing different types of sensing materials and working both as single selective sensors and/or in a multisensory arrangement for POPs compounds detection have been summarized and are listed in Table 1. The application of optical sensors in combination with the technological advancements of familiar electronic devices, such as smartphones, etc., open new perspectives for the development of effective, inexpensive, and user-friendly sensing platforms for concrete application tasks, including POPs monitoring and quantitative analysis.

**Table 1:** Optical chemical sensors for POPs detection.

Sensitive Material	Principle	Analyzed Compound	Application	Detection Limit	Concentration Range	Ref.
**Single Fluorimetric and Colorimetric Sensors**						
UCNPs@COFs	Fluorescence quenching	PFOS	Tap water and food packing	0.15 pM	0.18 pM–18 nM	[16]
NCDs	Fluorescence enhancement	PFOS	River and lake water	0.3 nM	3 × 10^−10^–1.6 × 10^−8^ M	[17]
UMIR probe	Fluorescence quenching (ratiometric)	PFOS	Lake water, human serum, egg	1 pM	0.001–0.1 nM	[18]
					0.1–1 nM	
GC5A-6C	Fluorescence enhancement	PFOS	Tap water, lake water	30 nM (PFOS)	0–2 μM	[19]
GC5A-12C		PFOA		39 nM (PFOA)		
BowtieCyclo	Fluorescence enhancement	PFOS	In water	47.3 nM	0–0.6 μM	[20]
Water-soluble copolymers based on thymine	Fluorescence enhancement	PAHs (BaP, Pyr)	Tap, ditch, and river water samples	0.11 ng/mL (BaP);	0.0–2.0 ng/mL (BaP)	[23]
				0.06 ng/mL (Pyr)	0.0–1.25 ng/mL (Pyr)	[24]
γ cyclodextrin-dye complex	Fluorescence enhancement	PAHs and metabolites	Human breast milk	0.32–59.52 μM	-	[25]
Ln-MOF	Fluorescence quenching	Polychlorinated aromatic compounds	-	-	0–1000 nM	[27]
BT-CTF	Fluorescence quenching	Primary aromatic amines (PAAs)	-	11.7 nM (PA);	3–33 μM	[28]
				1.47 nM (PDA);		
				26.2 nM (NPA)		
ssDNA/L-cysteine capped ZnS QDs/GO sheets	Fluorescence quenching	Edifenphos (EDI)	In laboratory	0.13 μg/L	0.5–6 μg/L	[29]
ZnO QDs@APTES	Fluorescence quenching	Aldrin, tetradifon, glyphosate, atrazine	In laboratory	-	-	[30]
PLNPs	Fluorescence quenching	Nitrofurazone	Milk and lake water	5 nM; 10 nM (TNP)	-	[31]
PDI derivatives	Fluorescence quenching	Berberine chloride	Commercial medicine	28 nM	1.0–30.0 μM	[32]
PDI derivatives	Fluorescence quenching	Polymyxins B	Meat	18.5 nM	1–2000 nM	[21]
BA–LMOFs@MIP	Fluorescence enhancement (363 nm)/quenching (618 nm)	Ribaverin	Eggs and lake water	7.62 ng/mL	25–1200 ng/mL	[34]
3,3,5,5-tetramethylbenzidine (TMB)/MoS_2_ -Fe_3_O_4_ nanocomposite	Colorimetric (Absorbance)	PFOS	-	4.3 ppb	0.05–6.25 ppm	[36]
Ethyl violet, ethyl acetate	Colorimetric (RGB)	PFOAPFOS	On field—real water samples	10 ppb (Dual LPE);	10–1000 ppb	[37]
				0.5 ppb (SPE)		
Toluidine blue TB	Dual-channel sensor: RRS (Rayleigh scattering) and colorimetric	PFOS	Real water samples	4.2 (nmol/L)	0.04–20.0 (μmol/L)	[38]
α,α,α,α-5,10,15,20-tetra-(2-amido-phenyl-pentadecafluoro-octanoyl)porphyrin	Colorimetric (Absorbance)	PFOA	Spiked soil sample	3 ppm	3–30 ppm	[39]
**Surface Enhanced Raman Spectroscopy (SERS) sensors**						
Microporous silica capsule/Au plasmonic films	SERS	DDT	Natural water	-	1 ppb–3 ppm	[58]
Ag NPs/non-woven fabric	SERS	several pesticides residues	In fruits	-	-	[59]
Nanoporous silver sheet	SERS	Organochlorine, as lindane	In laboratory	87 ppb	87–364 ppb	[60]
Au concave Nanocrystals	SERS	Lindane	In laboratory	-	30–300 ppm	[61]
MoO_2_	SERS	Bisphenol A (BPA), dichloropheno (DCP), pentachlorophenol (PCP)	In laboratory	10^−7^ M	10^−7^–10^−4^ M	[62]
AgNPs modified with organic p -acceptor molecules	SERS	PAH/PASH	In laboratory/fuel samples	Up to 10 × 10^−9^ M	0.05 × 10^−6^–50 × 10^−6^ M	[63]
Ag nanocubes/GO/AuNPs	SERS	Thiram, thiabendazole	Drinking-water	0.37–8.3 ppb	0.1–10 nM	[64]
AgNPs	SERS	DBT	In laboratory/	10^−6^ M	10^−5^–10^−3^ M	[65]
			petrol samples			
AgNPs	SERS	Organochlorine pesticides	In laboratory	10^−5^ M	-	[66]
Au nanosheets/	SERS	HCH	In laboratory	0.3 ppb	10^−9^ M–10^−5^ M	[67]
4-MBPA						
AuNPs/cysteamine	SERS	PCP	In laboratory	0.26 mg/L	1 nM–100 μM	[68]
AuNPs/HS-b-CD	SERS	anthracene, naphthalene	In laboratory	1 ppb/10 ppb	1 ppb–1 ppm	[69]
AuNPs/DSNB	SERS	benzo[a]pyrene	In laboratory	2 nM	-	[50]
AuNPs	SERS	PAHs	In laboratory	0.45 µg/L (PYR);	0–100 µg/L	[70]
				0.23 µg/L (PHE);		
				1.38 µg/L (NaP)		
AgNPs on Al_2_O_3_ nanotips	SERS	Ractopamine	Raw pork	10 µg/L	1.0 × 10^−8^–1.0 × 10^−4^ M	[71]
Ag@Fe_3_O_4_@Ag/β-CD NPs	SERS	BBP	In laboratory/liquor	1.3 mg/kg	5 × 10^−8^–5 × 10^−5^ M	[72]
Citrate-coated AuNPs	SERS	Chlordane	In laboratory/	1 ppm	0.5 ppm–10 ppm	[73]
			crude oil			
AgNpS	SERS	PAHs	In laboratory	5 ng/L (pyrene)	0–40 ng/L	[74]
				50 ng/L (benzo[a]pyrene)		
				100 ng/L (anthracene)		
Au-Ag alloyed nanocrystal/ZIF8	SERS	HCH	In laboratory	<1.5 ppb	5 × 10^−9^–1 × 10^−4^ M	[75]
Au-Ag/Si nanoporous—ZIF8	SERS	PCP	In laboratory	10^−13^ M	10^−13^–10^−7^ M	[76]
Au-MOF-5	SERS	Paraoxon, fenitrothion	In laboratory	<10^−12^ M	10^−14^–10^−6^ M	[77]
**Biosensors**						
Aptamers on AuNPs	Colorimetric	PCB 77	On field—real water samples	0.05 nM	0.5–900 nM	[80]
	biosensor					
Aptamers/cDNA and magnetic microspheres	Fluorescence	PCB72/106	On field	0.0035 ng/mL	0.004–800 ng/mL	[83]
	biosensor					
(LC)-based aptamer	Color intensity	PCB77	Food quality assessment	1.5 × 10^−5^ μg/L	1.5 × 10^−5^–15 μg/L	[84]
	biosensor					
Genetically engineered CSH cell	Luminescence cell biosensor	PBDEs	In laboratory	0.01 µM	0.05–6.0 µM	[88]
Mono-specific antibodies	SPR–POF biosensor	PFOA/PFOS	In laboratory	0.21 ppb	0 -100 ppb	[92]
**Photonic crystal (PhC) sensors**						
Poly(styrene-acrylic acid) and TiO_2_ NPs	PhC, naked-eye or smartphone detection	Benzene (benz), toluene (tol), xylene (xyl), 1,2,4-trimethylbenzene (TMB)	In laboratory	1.69 g/m3/410.9 ppm (tol);	0.0–81.18 g/m^3^ (tol);	[95]
				5.26 g/m^3^/1511.5 ppm (benz);	0.0–300 g/m^3^ (benz);	
				0.47 g/m^3^/99.2 ppm (xyl);	0.0–40 g/m^3^ (xyl);	
				0.079 g/m^3^/14.7 ppm (TMB)	0.0–14 g/m^3^ (TMB)	
Silicon slab	Hexagonal structured PhC	DDT and PCB	Drinking water	-	RI–1.5795 (DDT);	[96]
					RI–1.491; (PCB)	
**Surface plasmon resonance (SPR) sensors on** **Plastic optical fibers (POFs) and P** **hotonic crystal fibers (PCF)**						
SPR–POF-MIP	SPR	PFOA; PFOS; mixture of 11 perfluorinated alkylated substances (PFAs, C4–C11 range)	In water	0.13 ppb (PFOA);	0–4 ppb	[102]
				0.15 ppb (PFAs)		
D-shaped POF-SPR	SPR	PFOA	In water	0.5 ppb	0–200 ppb	[103]
Dual- channel solid silica/Au external coating	PCF-SPR	Compounds with	In laboratory	-	-	[104]
		RI 1.30–1.40				
Au/TiO_2_ thin film on glass	PCF	Compounds with	In laboratory	-	-	[105]
		I 1.33–1.41				
**Multisensor systems**						
CTAB encapsulated Cu NCs	Fluorescence quenching	Dithiocarbamates (DTCs)	On field	0.63 mg/L	1–100 mg/L	[108]
					Sensitivity 1.158 mg/L	
Fluorescent QDs	Fluorescence	NACs—nitroaromatic compounds	-	-	Classification: 13 NAC (0.1 mM)	[109]
	Optical filter					
LMOFs	Luminescence quenching	PFASs	-	-	Classification: 6 PFASs, (2 µg/mL)	[110]
CD + PVA	Luminescence	PAHs		0–200 μM	Classification: 16 PAHs (10 μM)	[111]
	Inner filter					
Conjugated polymers (CPs)	Fluorescence	Azo dyes	-	-	Classification:12 azo dyes (500 nM) in river—wastewaters6 azo dyes (15 μM) in seawater	[112]
	Inner filter					
Acetylcholinesterase (AChE), butyrylcholinesterase (BuChE)	Fluorescence	Organophosphourus (OPs) and carbamate pesticides	-	0–5 ppm	Classification: 30 OPs and carbamate up to concentrations equal to 0.2 ppm	[113]

## Data Availability

Not applicable.

## Abbreviations

4-MBPA	4-mercaptophenylboronic acid
AIE	aggregation-induced emission
Ala	alanine
ANHCs	Au nanosheets-built hollow sub-microcubes
APTES	3-aminopropyltrimethoxysilane
Asp	aspartic acid
BA-LMOFs	boric acid-functionalized lanthanide metal organic frameworks
BaP	benzo[a]pyrene
BBC	berberine chloride
BBP	benzyl butyl phthalate
BC	BowtieCyclo
BPA	bisphenol A
BT	benzothiadiazole
CA	cluster analysis
CDs	carbon dots
ChE	cholinesterase
CHS	cell surface hydrophobicity
COFs	covalent organic frameworks
CPs	conjugated polymers
CTCs	charge–transfer complexes
CTF	covalent triazine framework
Cys A	cysteine A
DBT	dibenzothiophene
DCP	dichlorophenol
DDQ	2,3-dichloro-5,6-dicyano-1,4-benzoquinone
DDT	dichloride diphenyl trichloroethane
DFT	density functional theory calculations
DMF	N,N-dimethylformamide
DSNB	(5,5 -dithiobis(succinimidyl-2-nitrobenzoate)
DTCs	dithiocarbamates
EDI	edifenphos
EDMA	ethylene glycol dimethacrylate
FOCS	fiber optic chemical sensors
FPO-NH4	ammonium perfluorooctanoate
FRET	fluorescence resonance energy transfer
Glu	glutamic acid
GO	graphene oxide
HCA	hierarchical cluster analysis
HCH	hexachlorocyclohexane
HCR	hybridization chain reaction
HS-b-CD	mono-6-thio-b-cyclodextrin
ICT	intramolecular charge transfer
IDA	indicator displacement assay
IFE	inner field effect
IIDA	intramolecular indicator displacement
LC	liquid crystal
LDA	linear discriminant analysis
LLE	liquid–liquid extraction
LOD	limit of detection
MIP	molecularly imprinted polymer
MMPs	magnetic microspheres
MNP	iron oxide nanoparticle
MOFs	metal-organic-frameworks
MQD	MoS_2_ quantum dots
NACs	nitroaromatic compounds
NCDs	nitrogen-doped carbon dots
NIP	non-imprinted polymer
NpAg	nanoporous silver
OPs	organophosphorus compounds
PAAs	primary aromatic amines
PAHs	(polycyclic aromatic compounds)
PARAFAC	parallel factor analysis
PASHs	(polycyclic aromatic sulfur-containing hydrocarbons)
PBDES	polybrominated diphenyl ethers
PCA	principal component analysis
PCB	polychlorinated biphenyls
PCF	photonic crystal fiber
PCP	pentachlorophenol
PDI	perylene diimide
PET	photo-induced electron transfer
PFAS	poly-fluoroalkyl compounds
PFCs	perfluorinated compounds
PFDA	1H,1H,2H,2H-perfluorodecyl acrylate
PFOA	perfluorooctanoic acid
PFOS	perfluorooctanesulfonic acid
PhC	photonic crystal
Phe	phenanthrene
PL	photoluminescence
PLNPs	persistent luminescence nanoparticles
PMB	polymyxins B
PMMA	poly(methyl methacrylate)
POPs	persistent organic compounds
Pyr	pyrene
QDs	quantum dots
RBV	ribaverin
RI	refractive index
SDBS	sodium dodecyl benzene sulfonate
SEM	scanning electron microscopy
SERS	Surface Enhanced Raman Spectroscopy
SLE	solid–liquid extraction
SPP	surface plasmon polariton
SPR–POF	surface plasmon resonance optical fiber
SPR	surface plasmon resonance
TCB	trichlorobenzene
TCh	thiocholine
TCP	trichlorophenol
TCPP	tetrakis(4-carboxyphenyl)porphyrin
TEM	transmission electron microscopy
THMS	triple helix molecular switch
TMB	1,2,4-trimethylbenzene
TNP	2,4,6-trinitrophenol
TPE	tetra phenylethylene
UANNs	urchin-like Au-Ag alloyed nanocrystal
UCNPs	upconversion nanoparticles
VBT	(vinylbenzyl)trimethylammonium chloride
VOCs	volatile organic compounds
WQD	WS2 quantum dots
ZIF-8	zeolite imidazole framework
ZnO	zinc oxide
